# SpikeShip: A method for fast, unsupervised discovery of high-dimensional neural spiking patterns

**DOI:** 10.1371/journal.pcbi.1011335

**Published:** 2023-07-31

**Authors:** Boris Sotomayor-Gómez, Francesco P. Battaglia, Martin Vinck

**Affiliations:** 1 Donders Centre for Neuroscience, Department of Neurophysics, Radboud University Nijmegen, Nijmegen, Netherlands; 2 Ernst Strüngmann Institute (ESI) for Neuroscience in Cooperation with Max Planck Society, Frankfurt, Germany; 3 Donders Institute for Brain, Cognition and Behaviour, Radboud University Nijmegen, Nijmegen, Netherlands; Université Paris Descartes, Centre National de la Recherche Scientifique, FRANCE

## Abstract

Neural coding and memory formation depend on temporal spiking sequences that span high-dimensional neural ensembles. The unsupervised discovery and characterization of these spiking sequences requires a suitable dissimilarity measure to spiking patterns, which can then be used for clustering and decoding. Here, we present a new dissimilarity measure based on optimal transport theory called SpikeShip, which compares multi-neuron spiking patterns based on all the relative spike-timing relationships among neurons. SpikeShip computes the optimal transport cost to make all the relative spike-timing relationships (across neurons) identical between two spiking patterns. We show that this transport cost can be decomposed into a temporal rigid translation term, which captures global latency shifts, and a vector of neuron-specific transport flows, which reflect inter-neuronal spike timing differences. SpikeShip can be effectively computed for high-dimensional neuronal ensembles, has a low (linear) computational cost that has the same order as the spike count, and is sensitive to higher-order correlations. Furthermore, SpikeShip is binless, can handle any form of spike time distributions, is not affected by firing rate fluctuations, can detect patterns with a low signal-to-noise ratio, and can be effectively combined with a sliding window approach. We compare the advantages and differences between SpikeShip and other measures like SPIKE and Victor-Purpura distance. We applied SpikeShip to large-scale Neuropixel recordings during spontaneous activity and visual encoding. We show that high-dimensional spiking sequences detected via SpikeShip reliably distinguish between different natural images and different behavioral states. These spiking sequences carried complementary information to conventional firing rate codes. SpikeShip opens new avenues for studying neural coding and memory consolidation by rapid and unsupervised detection of temporal spiking patterns in high-dimensional neural ensembles.

## Author summary

Neuronal coding and memory formation depend on temporal activation patterns spanning high-dimensional ensembles of neurons. With new recording technologies like Neuropixels, it has now become possible to measure from > 1000 neurons simultaneously. This raises the problem, how we can detect temporal sequences of spikes in these high-dimensional ensembles in an unsupervised manner. Here, we present a new method to solve this problem based on optimal transport theory, called *SpikeShip*. SpikeShip is a fast method for the unsupervised discovery of spiking patterns in high-dimensional data, and provides a principled measure of dissimilarity of multi-neuron spiking sequences. SpikeShip can be effectively computed for high-dimensional neuronal ensembles, has a low (linear) computational cost that has the same order as the spike count, and is sensitive to higher-order correlations. We apply SpikeShip to high-dimensional neural data to study the encoding of behavioral states and visual information by temporal spiking sequences. SpikeShip opens new avenues for studying neural coding and memory consolidation by rapid and unsupervised detection of temporal spiking patterns in high-dimensional neural ensembles.

## Introduction

Information in the brain is encoded by very high-dimensional “ensembles” of neurons, which encode information with spikes. Populations of neurons can produce specific spike patterns depending on sensory inputs or internal variables [1–8]. With new recording techniques like Neuropixels [9], it has become possible to simultaneously record from thousands of single neurons [10–12]. This offers new opportunities to uncover the relationship between multi-neuron spiking patterns and sensory inputs or motor outputs, yet also poses unique mathematical challenges for the unsupervised discovery of the “dictionary” of neuronal “code-words”.

The notion of information encoding relies on the construction of a distance or dissimilarity measure in an N-dimensional space. For example, the distance between binary strings can be measured using the Hamming distance. In the brain, the distance between two multi-neuron spiking patterns is conventionally based on differences in the firing rates (spike / sec). Using this method, it has been shown for example that high-dimensional neural ensembles span a low-dimensional manifold that relates to a stimulus or behavioral variables in a meaningful way [13, 14]. However, firing rates do not capture the potentially rich information contained by the precise temporal order in which spikes are fired, e.g. neuron *i* firing at time *t* and neuron *j* firing at *t* + *τ*. For instance, we expect that any time-varying sensory stimulus or action sequence may be encoded by a unique multi-neuron temporal pattern of spiking. Indeed, multi-neuron temporal sequences can encode information about sensory stimuli and are required for the generation of complex motor patterns like bird songs [1–7, 15, 16]. Temporal sequences may also be critical for memory formation, because neural plasticity rules are highly sensitive to the temporal order in which spikes are fired [17–21]. It is plausible that much of the information contained in spiking sequences has thus far not been discovered, as temporal correlations have typically been studied based on relatively small neural ensembles, whereas the number of pairwise spike-time relationships scales with *N*^2^.

A major computational problem is thus to measure the dissimilarity of spiking patterns in terms of the relative spike timing between neurons. Developing such a measure has several challenges, including 1) Techniques that rely on binning spikes and require exact matches of patterns (e.g. information theoretical measures) have several disadvantages: They require a relatively large number of observations due to combinatorial explosion, lack robustness against spike time jitter and reduce temporal resolution due to binning. 2) Computational cost becomes a major constraint for high-dimensional ensembles of neurons, and a measure should ideally have a computational cost that is linear in the number of neurons and spikes.

Here, we develop a novel dissimilarity measure for multi-neuron spiking patterns called SpikeShip, which has linear computational complexity of O(N), and has the key advantage of being sensitive to higher-order structures. SpikeShip can be interpreted as the optimal transport cost to make all spike-timing relationships between two different multi-neuron spiking patterns identical. That is, it solves the optimal transport problem for the entire spiking pattern, and yields a unique decomposition of spike pattern dissimilarity in terms of neuron-specific flows (which controls similarities in terms of relative spike times) as well as a global flow term (which controls the similarity in terms of absolute time). We demonstrated the power of the SpikeShip measure by applying it to large scale, high-dimensional neural ensembles in mice from [12] and [22], and demonstrating that temporal spiking sequences reliably distinguish between natural stimuli and different brain states. We discuss the properties of this measure compared to previous spike train measures like Victor-Purpura Distance (VP) [23, 24], SPIKE [25], and Rate-Independent SPIKE (RI-SPIKE) [26]. Finally, we show that SpikeShip carries orthogonal information compared with the traditional firing rates code.

## Results

Our overall goal is to develop a dissimilarity measure between multi-neuron spiking patterns that is exclusively based on the temporal order of spiking. Suppose that we have measured the spikes from *N* neurons (“spike trains”), with an average of *n* spikes divided into *M* epochs of length *T* (measured in seconds or samples). Epochs could be defined by e.g. trials (e.g. stimulus presentations) or sliding windows. The problem is to find a dissimilarity measure with the following properties:

1. The measure should depend on the temporal order of firing across neurons, but not on the spike count. We note that this point does not imply that spike count differences do not matter, but rather that it is often desirable to have a measure that can be strictly interpreted in terms of temporal sequences.
2. If two spike patterns are identical in terms of cross-neuron spike timing relationships (i.e. they are a temporally translated version of one another), then the dissimilarity measure should equal zero.
3. The measure does not require binning or smoothing and is based on the exact timing of the spikes.
4. It should measure dissimilarity in a gradual way, and avoid the problem of “combinatorial explosion” that occurs with methods that search for exact matches in spiking patterns. Combinatorial explosion means that for a very large number of neurons, the probability of an exact match in spiking patterns becomes extremely small.

**Algorithm 1:** SpikeShip distance computation of population spike time vectors between two epochs.

**Input**: Spike times vectors of *N* neurons for epochs *k* and *m* (*st_k_*: array, *st_m_*: array)

**output**: Pair of total neuron-specific distance and global shift (*f*: double)

**Function**: *SpikeShip*(*st*_*k*_, *st*_*m*_) :

 Initilize *c*, *w*, $A_{km}$ (arrays), and $n_{i}^{*}$ (integer);

 **for** *i* ∈ {1, …, *N*} **do**

  **if** len(*st*_*k*_[*i*])> 0 ***and*** len(*st*_*m*_[*i*])> 0 **then**

   Store costs (*c*_*i*_) and weights (*w*_*i*_) from EMD(*st*_*k*_[*i*], *st*_*m*_[*i*]);

   Add *i* to $A_{km}$;

   Increase $n_{i}^{*}$ by len(*c*_*i*_)

  **end**

 **end**

 *g*^*min*^ = WeightedMedian(*c*, *w*);

$f=\frac{1}{\text{LEN}(A_{km})}\sum_{i\in A_{km}}\frac{1}{n_{i}^{*}}\sum_{u}^{n_{i}^{*}}w_{i,u}\left(\right|c_{i,u}-g^{min}\left|\right)$

 **return** *f*

**end**

**Fig 1:**
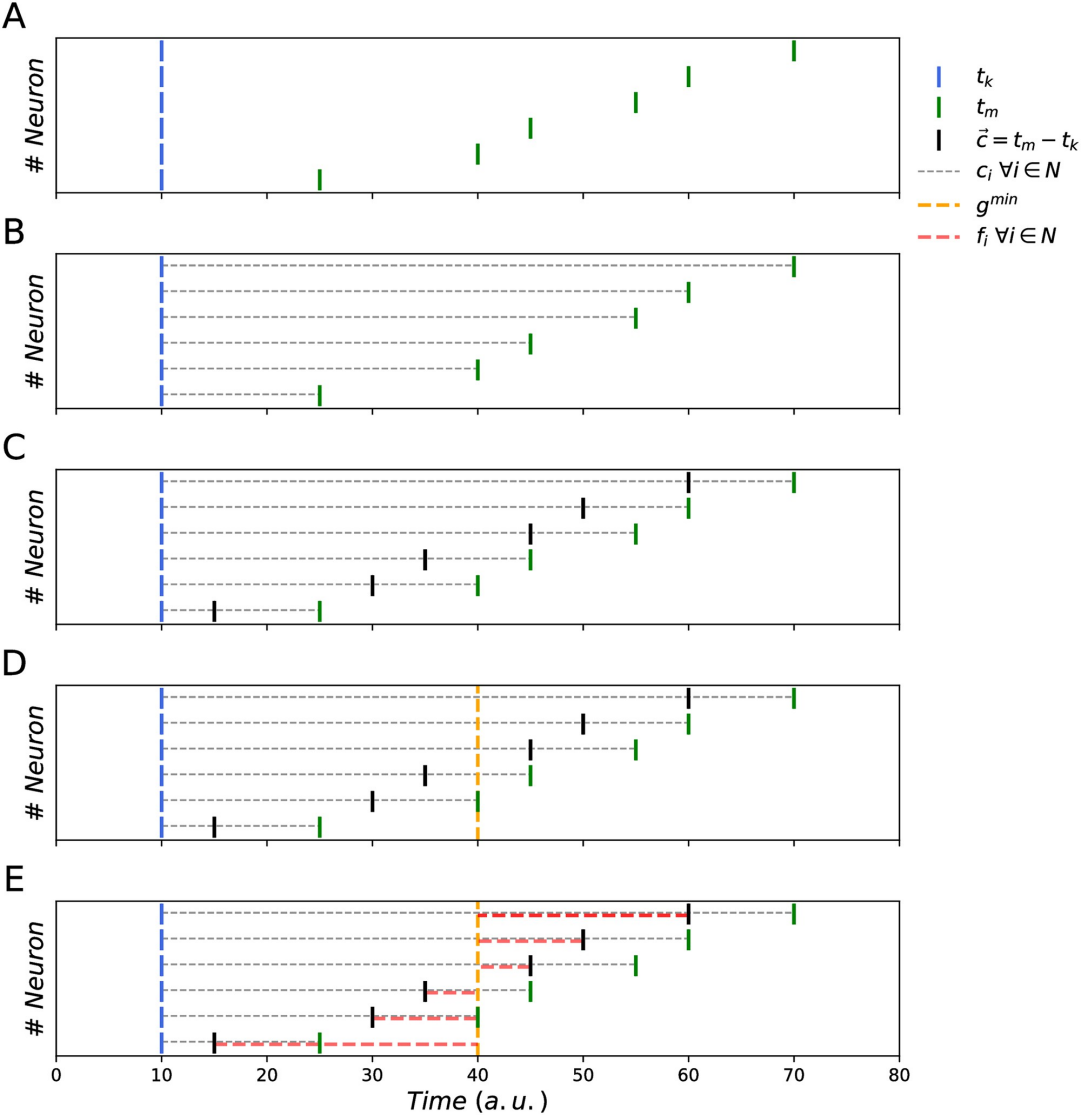
Illustration of SpikeShip. A) Example of two epochs with spike times *t*_*k*_ = (10, 10, 10, 10, 10, 10) and *t*_*m*_ = (25, 40, 45, 55, 60, 70) (note only one spike per neuron in this example). B) Distances between spike times *t*_*k*_ and *t*_*m*_. C) The vector $\vec{c}$ contains the differences of spike times *t*_*k*_ and *t*_*m*_. D) Computation of the median of $\vec{c}$: *g*^min^ = 40. E) *g*^min^ is the optimal global shift such that *f*_*i*_ = *c*_*i*_ − *g*^min^. The neuron-specific shifts $\vec{f}=(-25,-10,-5,5,10,20)$ contain all the information about the structure of distances between *t*_*k*_ and *t*_*m*_. SpikeShip equals $F_{km}=\frac{1}{6}\sum_{i}^{N}\left|f_{i}\right|=\frac{75}{6}$.

We introduce a measure that satisfies these constraints, called the SpikeShip measure (see Methods). The idea of SpikeShip is to measure the dissimilarity between spike trains using the mathematical framework of optimal transport, as shown in Figs 1 and S1. and pseudocode 1. We will consider each spike train as a collection of “masses” (i.e. the spikes). All spikes from each active neuron, together, contribute a unit mass, i.e. the mass of each spike is normalized to the total mass. This ensures the rate invariance of the method. The question now is what the optimal way is of transporting the masses in time to make the two patterns identical in terms of the relative spike times among neurons. In other words: The optimal transport problem is stated as finding the minimum cost of shifting the (unit) mass of the spike train in each neuron in epoch *k*, such that the cross-correlations (i.e. sequential firing) between all of the neurons become identical to those in another epoch *m*. Solving this problem yields a dissimilarity measure that is strictly defined based on *relative* timing among neurons, i.e. not on the absolute timing of the spikes.

Intuitively, one would suspect that measuring the similarity of spike train patterns based on the relative spike timing among *N* neurons has a computational complexity of at least order *N*^2^, which would make the method impractical for larger data sets. However, we surprisingly show that there is a fundamental solution that can be computed in order *N*.

We show that the global optimal transport problem can be solved in two steps:

1. We first compute the optimal transport flow to transform a spiking pattern in epoch *k* into the spiking pattern in epoch *m*, such that the patterns are identical in terms of *absolute* timing.
2. Transport cost is now minimized by computing a global temporal translation term and subtracting this term from all the individual spike shifts. This yields neuron-specific flows, and allows us to compute the total transport cost needed to make two patterns identical in terms of relative spike times.

*Step 1:* The algorithm starts by computing the Earth Mover Distance (see Methods, S1 Fig) for each neuron separately in step 1, shifting mass from each spike in pattern *k* to the spikes in pattern *m*. For instance, if the neuron has 2 and 6 spikes in pattern *k* and *m*, the associated masses would be (1/2, 1/2) and (1/6, ⋯, 1/6) respectively. Thus, the EMD distributes the 2 masses into 6 clusters with minimum transport cost. This effectively means that each of the two spikes is broken up into 3 parts which are then moved.

To make this more intuitive and simplify the analytical expression of SpikeShip, we note that breaking each spike into 3 parts is identical to replicating each spike 3 times (note that in the actual EMD algorithm, this replication is not performed). This amounts to finding the smallest common multiple of the spike counts for each neuron, denoted $n_{i}^{*}$ (see Methods), and replicating each spike $\frac{n_{i}^{*}}{n_{i,k}}$ and $\frac{n_{j}^{*}}{n_{i,m}}$ times. For instance, with spike counts *n*_*i*,*k*_ = 2 and *n*_*i*,*m*_ = 6 then the least common multiple equals 6, and spikes are replicated 3 and 1 times, respectively. Each spike will now have a weight of $w_{i}\equiv \frac{1}{n_{i}^{*}}$, such that the total mass is 1.

The EMD yields flows *c*_*i*,*u*_ (with unit time) for each *i*-th neuron and its *u*-th (replicated) spike ($1,\cdots ,n_{i}^{*}$). With flow we mean the temporal shift that is assigned to the spike. The moved mass (associated with the spike) is *w*_*i*,*u*_, such that ∑_*u*_*w*_*i*,*u*_ = 1.

*Step 2:* Importantly, the EMDs obtained from Step 1 only indicate the similarity of *absolute* spike times between two epochs, i.e. are based on the alignment of the spikes relative to an event. Yet, we stated that our measure should reflect the *relative* timing of spikes between neurons. We show that this can be achieved using the EMD flows computed in Step 1, by subtracting a global rigid translation term. This uniquely yields the minimum transport cost to transform the multi-neuron spiking pattern in epoch *k* such that its relative spike-timing relationships between neurons become identical to another pattern *m*. In the Methods section, we state our main theoretical result, namely that the optimal transport flows are given by

$$\begin{array}{c} f_{i,u}=c_{i,u}-g^{\text{min}}\;. \end{array}$$ (1)

Here *g*^min^ is the weighted median across all the original flows *c*_*i*,*u*_ with associated mass *w*_*i*,*u*_. Thus, we can decompose the transport flow in two terms: (1) an optimal global shift between two epochs, shared across all neurons; and (2) an optimal neuron-specific transport flow. We then define SpikeShip (see Methods) as

$$\begin{array}{c} F_{km}\equiv \frac{1}{\left|A_{km}\right|}\sum_{i\in A_{km}}w_{i,u}\sum_{u=1}^{n_{i}^{*}}\left|f_{i,u}\right|\;. \end{array}$$ (2)

Here $A_{km}$ is the set of all neurons that fired a spike both in epoch *k* and *m*. The weight $w_{i,u}=\frac{1}{n_{i}^{*}}$, effectively assigns an equal weight to each neuron that contains at least one spike, where $n_{i}^{*}$ is the (replicated) number of spikes.

The algorithm to compute SpikeShip has computational complexity O(Nn), because the weighted median has complexity O(Nn) (S2 Fig, and pseudocode 1). This means that SpikeShip performs much better in terms of computational complexity than previous measures like SPOTDis (which is $O(N^{2}n^{2})$) [27] and it thus becomes feasible to compute for large ensembles of neurons, as we will show further below.

**Fig 2:**
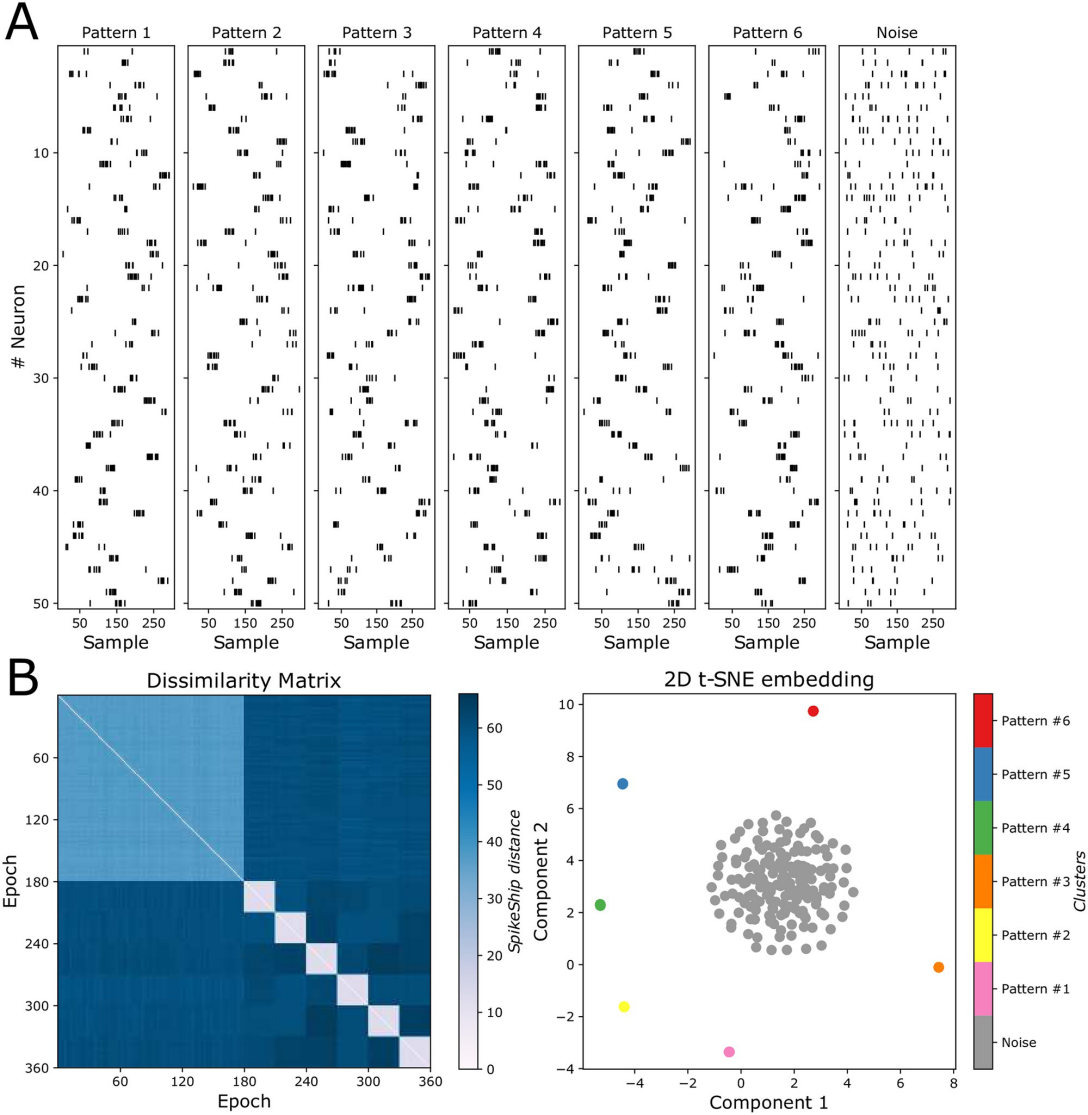
Dissimilarity matrices and clustering comparison. A) Six multi-neuron spiking patterns that were generated according to different inhomogenous Poisson processes, plus one noise pattern (homogeneous Poisson). We show the first 50 neurons from a total of *N* = 500 neurons. The first 180 epochs correspond to homogenous noise (random firing), and the next 180 correspond to pattern that were generated based on an inhomogenous Poisson process (6 different patterns with 30 epochs each). Each pattern was simulated with *T*_*epoch*_ = 300 samples, *T*_*pulse*_ = 30 samples. The firing rates outside the pulse were λ_*out*_ = 0.02 spks/samples and during the pulse λ_*in*_ = 0.2 spks/samples, respectively. The homogenous noise pattern had the same average firing rate as the inhomogeneous patterns. B) Sorted Dissimilarity matrix by pattern using SpikeShip dissimilarity measure (left), and a 2-dimensional t-SNE embedding using SpikeShip dissimilarity matrix (right).

Having computed a dissimilarity measure between multi-neuron spike trains, we can use embedding and clustering techniques to detect patterns in an unsupervised way. The rationale of our approach is that unsupervised clustering can be performed based on the dissimilarity matrices, rather than on the spike train data itself. To illustrate this, we generated 6 input patterns defined by the instantaneous rate of inhomogeneous Poisson processes, as in [27]. Noise was generated with random firing based on a homogenous Poisson process with a constant rate (i.e., homogenous noise) (See Fig 2A). We computed the pairwise distances between pairs of epochs using SpikeShip distance, yielding a dissimilarity matrix (Fig 2B). The patterns contained in the data can be visualized using manifold learning algorithms such as t-SNE, using the SpikeShip dissimilarity matrix as input [28–30] (Fig 2C). Furthermore, HDBSCAN [31] automatically detected clusters on the basis of the SpikeShip dissimilarity matrix. These results illustrate how SpikeShip can unveil multi-neuron spiking patterns and shows its efficiency in simulated, high-dimensional data.

An important property of SpikeShip is that it can distinguish spiking patterns even when they are multi-modal. To demonstrate this, we use multiple bimodal activation of Poisson patterns with patterned noise and homogeneous noise (See S3A Fig). In addition, we simulated a special case when neurons are “deactivated” in a certain segment of the epoch (See S3B Fig). We observed that, in both cases, SpikeShip can detect the patterns successfully and each cluster were well separated as shown in their dissimilarity matrices and 2D t-SNE embeddings. Finally, compared to the SPOTDis measure, which is also based on optimal transport (SPOTDis), we found that SpikeShip can detect patterns that are defined by lower signal-to-noise ratios (See S4 Fig).

### Properties of SpikeShip and comparison to other methods

**Fig 3:**
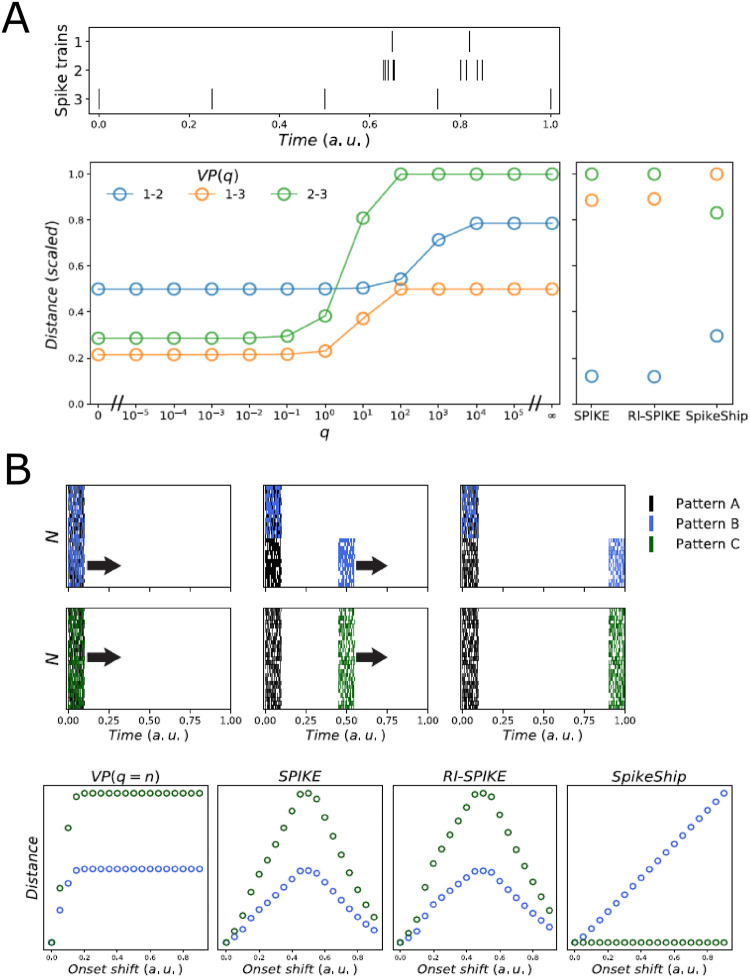
Comparison of metrics for example spike trains. A) Sensitivity to spike timing and spike count for different measures for an example of three synthetic spike trains (as in [32] (Fig 5D)). Bottom: Computation of Victor-Purpura metric for pairs of spike trains, varying the shift-cost *q* (left), and computation of distances for SPIKE, RI-SPIKE, and SpikeShip (right) (see Methods). SpikeShip and RI-SPIKE dissimilarities reflect spike timing rather than spike count. By contrast, VP reflects spike-count differences, which holds true for the entire range of *q*. B) Sensitivity of different measures to global shifts of spiking patterns and changes in relative spike timing. Top: Example of three multi-neuron spiking patterns. Onset of spike patterns *B* and *C* where shifted relative to pattern *A*. Colors correspond to the shift/delay applied to half (blue) and the full neural population (green). Bottom: Comparison between spike train dissimilarities between patterns (*A*, *B*) (blue) and pattern (*A*, *C*) (green).

Next, we show that, besides the properties discussed above, SpikeShip has several features that make it well-suited as a dissimilarity measure for spike train patterns based on relative timing relations, and that distinguish it from previous spike train metrics:

(1) SpikeShip measures the dissimilarity of spiking patterns between a pair of epochs based on the *relative* spike timing between neurons. SpikeShip can therefore be used to detect patterns that are spontaneously occurring or that are not locked to the onset of an event, using e.g. sliding window approaches. By contrast, other measures like VP and SPIKE are not based on the relative spike timing. VP and SPIKE can, however, be used to compare spike trains in different epochs based on the *absolute* spike timing (Fig 3B). In this case, these measures are computed for each neuron separately by directly comparing the spike trains in different epochs, one neuron at a time.

To show that SpikeShip can be used in cases where the onset of the pattern was not known, we performed two kinds of simulations.

First, we studied a case where the onset of the patterns was not known, and the duration of the pattern was also not known. In this case, a sliding approach could be used to optimize the window length based on Silhouette score. We found that SpikeShip can be effectively used with a sliding window approach, as shown in S5B Fig, in contrast to SPOTDis and Victor-Purpura.

Second, we considered a scenario in which there were random global shifts superimposed onto different patterns (See S6A Fig). Here, we added simulations in which there were different patterns, plus global shifts of these patterns relative to the epoch onset. Importantly, the global shifts were not systematically related to the different patterns. As shown in S6B Fig, VP is influenced by these global shifts, which reflects the fact that it measures differences in absolute timing. By contrast, SpikeShip still detects the original patterns based on relative spike timing. In addition, SpikeShip yields the global flow term and thereby directly provides a global measure of latency of the entire pattern.

(2) SpikeShip has a linear dependence on differences in relative spike-timing between epochs (Fig 3B). Conversely, because VP has a hyperparameter (*q*), it can have a highly non-linear dependence on spike timing. This is because as differences in timing become larger, VP is exclusively driven by insertions, such that the total cost does not increase (Fig 3B).

We further observed that the two other measures, SPIKE and RI-SPIKE, were also influenced by the distance of spiking patterns to the edge of the window (Fig 3B).

(3) By design, SpikeShip is only sensitive to timing relationships, and not to firing rates. The reason is that all the spike trains due to normalization have the same mass. By contrast, VP also reflects differences in spike counts because it also includes the insertion cost (See S7 Fig). In fact, VP will only reflect differences in spike counts when its hyperparameter *q* is very small or very large, and exhibits temporal sensitivity only for intermediate values of *q*. While SPIKE may have some rate sensitivity, RI-SPIKE is designed to have low rate sensitivity [26].

To illustrate these differences, we first examined an example previously shown in [32], with three patterns that either differ by firing rate (1 and 2) or by timing (1 and 2 vs. 3) (Fig 3A). In this example, the VP distance is primarily influenced by rate differences, irrespective of the choice of *q*. For all *q*, VP does not assign the lowest between patterns 1 and 2 despite these two patterns having a very close temporal relationship. By contrast, SpikeShip, SPIKE, and RI-SPIKE do assign a much lower cost between patterns 1 and 2 (See Fig 3A). Additionally, we noted that several measures require temporal alignment. To demonstrate this point, we simulated three Poisson patterns *A*, *B*, and *C*, as shown in Fig 3B. Here, the pattern *A* contains Poisson spikes in a specific interval of the window length. Also, we generated the same amount of patterns but after applying a linear shift for half of the neural population and the entire neural population (pattern *B* and *C*, respectively) as illustrated in Fig 3B. We found that SPIKE and RI-SPIKE are affected by the position of the stimulus onset. Additionally, VP assigns a maximum distance when the cost of shifting spikes is greater than the cost to insert them. Thus, these measures require a clear definition of the stimulus onset to cover as the definition of the window length affects their distances.

Second, we studied different spiking sequences together with a local or global scaling of firing rates across epochs. As shown in S7 Fig, VP distance did not uniquely distinguish the patterns based on temporal or rate structure, and is mainly influenced by differences in firing rates for a large range of *q*. By contrast, SpikeShip was not influenced by the scaling of firing rates between epochs (See S8 and S9 Figs). Finally, both SPIKE and RI-SPIKE were less effective than SpikeShip as they assign very high dissimilarities between the noise spike trains, such that the overall clustering performance was worse than for SpikeShip (See S10 Fig).

We emphasize that the crux of SpikeShip is that it effectively measures dissimilarity in terms of *cross-neuron* spike-timing relationships, rather than absolute time. Nevertheless, the first step of the algorithm is to shift spikes only for the same neuron between epochs. However, this is only a computational trick to arrive at the cost based on inter-neuronal spike-time relationships, which is obtained by subtracting the global weighted median. In the Methods section, we show that this yields a measure that effectively measures the dissimilarity in terms of inter-neuronal spike time relationships, rather than in absolute time.

### Application to Allen Brain Institute’s neural datasets

Next, we applied SpikeShip to several high-dimensional neural datasets. Previous work has shown that the visual system can very rapidly process natural images and extract categorical information [33]. It has been proposed that this relies on a temporal coding strategy, whereby visual information is encoded based on the temporal sequence of spikes relative to stimulus onset [33].

**Fig 4:**
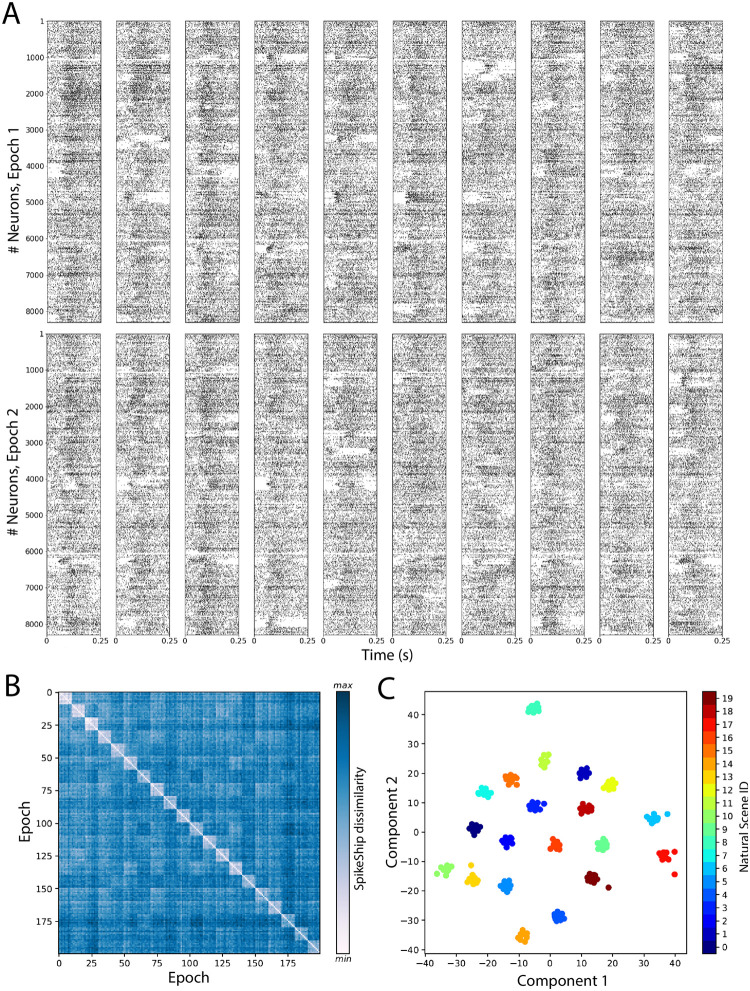
SpikeShip analysis of spike sequences for natural scenes presentations (Allen Brain Institute). A) Raster plot of two epochs with *N* = 8, 301 neurons for each presentation. B) Sorted dissimilarity matrix by image ID for 20 natural scenes presentations with 10 presentations each (*M* = 200). C) 2-dimensional t-SNE embedding for each presentation.

Here, we used SpikeShip to determine whether temporal spiking sequences in six visual areas can reliably distinguish natural images from each other. To this end, we analysed Neuropixel data from 32 mice while they passively viewed natural images (dataset from the Allen Institute for Brain Science; see Methods). A total of 20 natural scenes were selected with 10 repetitions each (i.e. *M* = 200 epochs). To create a high-dimensional vector of neurons, we pooled together all recorded neurons (*N* = 8, 301) across the 32 mice (See Fig 4A). Note that we pooled data across recording sessions to demonstrate that our method is computationally scalable to very large number of neurons. In S11 Fig we also present an analysis of individual sessions (i.e. without pooling data across mice).

The pairwise SpikeShip distance is presented in Fig 4B as a dissimilarity matrix sorted by the presentations of each natural scene. The t-SNE embedding revealed clear clustering of spiking patterns based on SpikeShip, such that different natural images could be reliably distinguished from each other (See Fig 4C). Hence, natural images yielded distinct temporal spiking sequences that were time-locked to stimulus onset (so that they could be extracted from combined data from multiple sessions), in support of the idea that the visual system may use a temporal coding strategy. In sum, these findings demonstrate that SpikeShip can unveil multi-neuron temporal spiking patterns from high-dimensional recordings.

### Comparison of SpikeShip vs firing rates in visual stimuli

**Fig 5:**
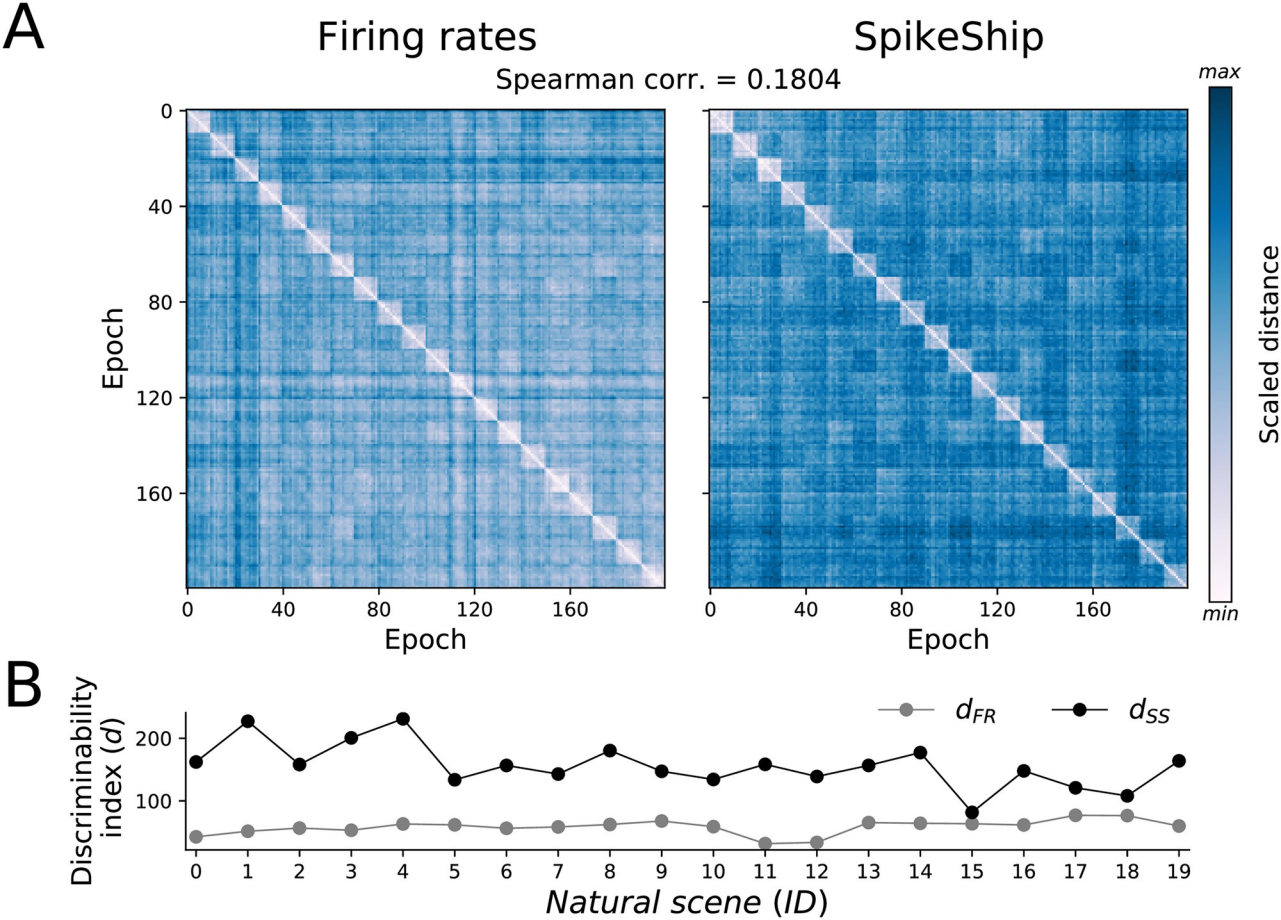
Comparison of pairwise distances between Firing rates and SpikeShip. A) Dissimilarity matrices for Allen Brain Institute data (sorted by natural scenes). Colormaps were changed to show more variability in distances. B) Discriminability index for dissimilarity matrices shown in A) by using firing rates (*d*_*FR*_) and SpikeShip (*d*_*SS*_).

We wondered how the information in temporal spiking sequences compared to the information carried by conventional firing rate codes. Our first question was, which one of these two codes conveyed more information. To determine this, we computed a distance matrix for the firing rates, by computing the Euclidean distance between the firing rate vectors. These distances were computed in the same time window as we used for SpikeShip (Fig 5A).

We then computed a measure of discriminability between natural images based on the distances within and between images (see Methods, Eq 29). We found that there was a higher discriminability between natural images for SpikeShip as compared to firing rate vectors for all the natural scenes (Fig 5B).

Next, we wondered to what extent the information in SpikeShip was independent of the firing rate information. To this end, we computed the Spearman correlation between the SpikeShip distance and firing rate dissimilarity matrices, which contained information from all epochs across all natural images. We found that the dissimilarity matrices of SpikeShip and firing rates were only weakly correlated across epochs (Spearman correlation equals 0.1804). Accordingly, the t-SNE visualization shows that the relative locations of the clusters show major differences between both methods (S12 Fig).

Altogether, these results show that SpikeShip and firing rates contain different and to a large extent independent information about natural stimuli. Furthermore, SpikeShip allowed for a better separation of the different natural stimuli in comparison to firing rates. We further observed that both SPIKE and RI-SPIKE were less effective in separating the different patterns than SpikeShip, and showed a stronger correlation with firing rates (S13 Fig).

Finally, we performed analyses across single sessions. For the clustering analysis of natural scenes across single sessions, SpikeShip outperforms Rate-independent SPIKE measure (RI-SPIKE), as shown in S11 Fig. However, differences in clustering performance for SpikeShip vs. Firing rates did not reach significance, which may be due to a floor effect due to the lower ARI scores related to the smaller number of neurons.

These findings support the idea that the visual system may use spiking sequences to encode information about natural scenes, as proposed by e.g. [33].

### Application to spontaneous activity

Next, we applied SpikeShip to high-dimensional neural recordings from multiple brain areas while mice spontaneously transitioned between different behavioral states. Previous works have shown that behavioral states have major effects on the firing rates of neurons across multiple brain areas [10, 34]. Recent work has shown that different facial motion components outperform the prediction of firing rates as compared to running speed and pupil diameter (a measure of arousal). Hence, we wondered whether different behavioral states are accompanied and distinguished by specific spiking sequences.

**Fig 6:**
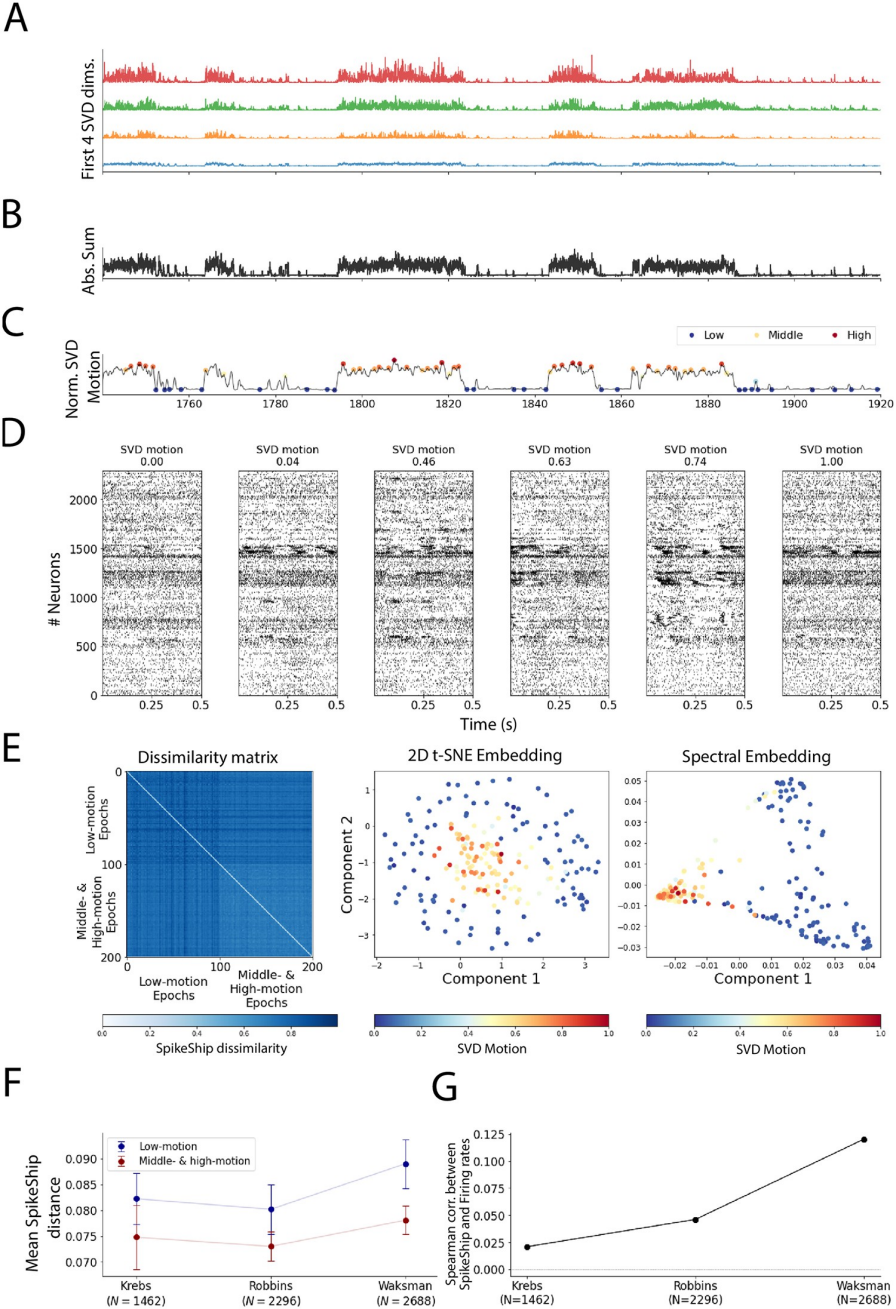
Analyses of multi-areal recordings during spontaneous activity. A) First 4 SVD dimensions of the face motion from the [22] data. B) Absolute sum of SVD dimensions shown in A) minus the median across such dimensions (denoising). C) Normalized SVD motion computed via a sliding window to smooth the whisker’s motion trace. We detected low- and middle- & high-motion states using HMM (See Methods). D) Raster plot for *N* = 2, 296 neurons for different epochs. E) Multi-spike sequence analyses. Left: Dissimilarity Matrices. Middle: 2D t-SNE embedding. Right: 2D Spectral Embedding (Laplacian Eigenmaps). F) Mean SpikeShip distance from [10] experiments. Error bars represent the standard deviation of SpikeShip distances. G) Spearman correlation between firing rates and SpikeShip per experiment.

To investigate this, we analysed the data set of [22], which contains multi-areal recordings from >1000 neurons in three mice. Similar to [10], we distinguished between different states based on the facial motion components using the SVD (singular value decomposition), and identified low, medium, and high-motion epochs (Fig 6A–6C). We randomly selected one hundred epochs for both low-, medium and high-motion states and computed the SpikeShip dissimilarity matrix (*M* = 200, See Fig 6D).

In Fig 6E, we show the dissimilarity matrix for one mouse (“Waksman”; *N* = 2, 688, for the other two mice see S14 Fig). The first 100 epochs represent low-motion states, and the remaining 100 are the middle- and high-motion epochs. The dissimilarity matrix reveals that the spiking sequences during medium and high motion are relatively similar to each other, whereas there is a relatively high variability among sequences during low-motion epochs (See Figs 6E and 6F and S15). Furthermore, both the t-SNE embedding and the spectral embedding show a separate state-space region for the spiking sequences during medium and high motion.

We further wondered if SpikeShip contains orthogonal information compared to firing rates, similar to what we had observed for natural images. Again, we computed the Spearman correlations between the SpikeShip and the firing rate dissimilarity matrices. We found relatively weak correlations between the firing rate and SpikeShip dissimilarity matrices for the three mice: (0.189, 0.024, −0.064) for *N* = (1462, 2296, 2688) neurons, respectively (See Fig 6G).

Altogether, these findings indicate that different brain states give rise to specific temporal correlation patterns across neurons, with relatively homogeneous spiking sequences during active behavior as compared to quiescence.

## Discussion

We studied the problem of measuring the dissimilarity between two multi-neuron spiking patterns based on the relative spike-timing relationships across neurons (i.e. firing order). We developed a new measure called *SpikeShip*. SpikeShip solves the problem of optimally transporting the spikes of individual neurons, such that the global pattern of spike-timing relationships becomes identical between two epochs. Intuitively, one would think that such a measure has a computational complexity of at least $O(N^{2})$, but we show that it can be computed with O(N) computational complexity; this is a major improvement over our previous work [27]. We show that a dissimilarity between two spiking patterns can be decomposed into neuron-specific flows and a temporal rigid translation term. Importantly, SpikeShip is not restricted to the 2nd order correlations, but is based on the higher-order structure in the spike train.

### Comparison to previous measures

We note that there are various other approaches to quantify multi-neuron patterns, which allow for different applications compared to SpikeShip. Some recent methods use dimensionality reduction and the identification of latent factors via non-negative matrix factorization or sparse convolutional coding, e.g. [35–38]. Although the dissimilarity matrices obtained via SpikeShip may in principle be used to identify latent factors, this remains to be explored in future work. Another approach is frequent-item-set mining, which looks for exact matches in spiking patterns across neurons (for an overview, see [39]). Finally, there are classic techniques based on statistical approaches that detect pairwise correlations between neurons at certain lags [39–41]. The idea of SpikeShip is not to examine whether neural data contains significant pairwise correlations. However, its fast computational time may make it useful to detect higher-order correlations by comparing observed spiking patterns with a surrogate distribution obtained via permutation (e.g. neuron identities across epochs).

Multi-neuron extensions of VP-distance [23, 42] and van Rossum distance [43] have been previously proposed. For instance, in case of [44], there is a hyperparameter *k* that quantifies the cost of exchanging the labels of a spike (i.e. which neuron it belongs to). In case *k* = 0, the multi-neuron metric amounts to (1) pooling all the spike trains into one spike train, and then (2) computing the VP distance. In this case, the precise inter-neuronal time-relationships are not considered, in contrast to SpikeShip. In case *k* ≥ 2, the multi-neuron distance amounts to computing the VP distance per neuron separately, and then averaging. We note that this is exactly how we computed the multi-neuron distance in case of VP. In case *k* ∈ (0, 2), it is expected that the multi-neuron VP distance has sensitivity both to inter-neuronal timing relationships and to single-neuron absolute timing. Given the nature of the multi-neuron VP, there are major differences with SpikeShip and the measures have essentially different applications. Most importantly, the multi-neuron VP metric is influenced by the absolute timing of individual neurons and global shifts of spike patterns, whereas SpikeShip is designed to measure dissimilarity in terms of inter-neuronal spike time relationships. SpikeShip also has some computational advantages compared to the multi-neuron VP extension: For the multi-neuron VP measure, the optimization problem is rather complex given two hyperparameters *q* and *k*. Finally, the multi-neuron algorithm has a computational cost that scale exponentially with the neural population size (i.e. $O(n^{N+1})$, where *n* is the average number of spikes and *N* the amount of neurons.

SpikeShip has several distinct properties as compared to other measures: 1) It is useful to note that there are different measures that are designed for distinct computational problems. SpikeShip is explicitly designed to measure the similarity between patterns in terms of relative spike time relationships. This distinguishes it from e.g. the VP distance, which measures similarity in terms of absolute spike times. We note that while SpikeShip is designed to measure similarity based on relative spike timing, it can also be used to measure similarity in terms of the absolute timing of spikes. In doing so, it has the advantage of extracting separately a global translation term, indicating shared latency shifts, and inter-neuronal timing differences. We furthermore showed that SpikeShip can be used in combination with a sliding window approach. Here the computational cost offers a great advantage as many different window lengths can be compared based on e.g. Silhouette score, as we show here. 2) SpikeShip is by design not sensitive to global or local scaling of firing rates, as opposed to VP. We also note that RI-SPIKE is designed to be insensitive to a scaling of firing rates. We note however that SpikeShip dissimilarity between any two epochs is based on the spike trains of those neurons that fire at least one spike. Thus, although the value of SpikeShip is not biased by firing rate, the firing rate can influence which neurons the measure is computed over, in particular when neurons have very low (baseline) firing rates. 3) SpikeShip finds sequences based on higher-order temporal structure based on relative spike timing, with a computational cost of O(Nn). By contrast, the previous SPOTDis measure [27] has computational cost of $O(N^{2}n^{2})$ and is strictly based on second-order correlations. 4) As shown here, SpikeShip is highly noise robust, and outperforms our previous method SPOTDis. 5) SpikeShip can be used for the detection of multi-modal patterns, which e.g. distinguishes it from methods that detect sequences based on the latency of firing. 6) SpikeShip is a bin-less measure, i.e. it is based on exact spike timings. 7) SpikeShip does not require exact matches between patterns, but is based on a metric distance (earth mover distance) that reflects the magnitude of differences in relative spike timings. This is an important difference relative to measures that e.g. require exact matches in patterns. Yet it also distinguishes it from e.g. VP, which shows a non-linear dependence on timing differences, and SPIKE and RI-SPIKE, which show a non-monotonic dependence on timing differences.

We note that SpikeShip has an efficient computation time of order *Nn* (number of neurons times number of spikes), which is comparable to the computational cost of the spike count. This is remarkable given that SpikeShip quantifies the dissimilarity based on all the relative spike-time relations. SpikeShip achieves this computation time by computing the Earth Mover Distance (EMD) first for each spike train separately, and obtaining individual flows by computing a global flow. We note that EMD was also applied to cross-correlations (in [27]) and to individual spike trains [45]. In [45] the EMD is quantified one neuron at a time, i.e. without considering inter-neuronal spike-time relationships, and the dissimilarity of spiking pattern is thus based on the absolute timing relative to a stimulus onset. Crucially, SpikeShip aims to quantify the dissimilarity of spiking patterns (between two epochs) in terms of the spike-timing relationships among *all* recorded neurons (i.e. inter-neuronal spike time relationships). This means that SpikeShip is based on relative spike-time relationships, which makes it invariant to e.g. the onset of a sequence relative to the beginning of an epoch, and allows for the quantification of spontaneously occurring sequences that are not locked to a stimulus onset. Thus, SpikeShip allows for a wide variety of applications (including sequences time-locked to a stimulus onset) and is thus more generic than EMD computed per neuron separately.

Finally, it is interesting to note that the SpikeShip algorithm can in principle also be used to align temporal sequences in an unsupervised way if there is a global jitter between spike patterns. The reason is that SpikeShip decomposes the transport cost between any two trials in terms of a global translation term (which is a non-linear computation) and neuron-specific shifts. Thus, SpikeShip can be used to achieve something similar as in a recent study [46], described as “time warping”.

### Application to neural data

We applied SpikeShip to large, real neuronal datasets of experiments in mice. We found that SpikeShip can be used for the unsupervised decoding of different natural images from a high-dimensional temporal spiking pattern across *N* > 8000 neurons. We note that for the main analyses we pooled data across mice, which therefore does not include shared trial-by-trial variability across neurons and only examines spike-timing patterns that are time-locked to stimulus onset. We found that the clustering performance of Spikeship outperforms the clustering based on firing-rate dissimilarity matrices, and that the spike timing information was only weakly redundant with the information in the firing rate vector. Note that although we measured clustering performance to the ground-truth, we did not directly compare classifiers based on SpikeShip vs. rate distances.

This suggests that spike timing information carries additional information relative to the firing rate, as has been hypothesized by [33]. Interestingly, the SpikeShip technique does not require the explicit identifications of spike latencies, and is able to extract higher-order correlations from spiking patterns and also distinguish multimodal patterns from each other [27]. Furthermore, as we showed here, the computation is extremely efficient.

In another application, we analysed large-scale recordings from the visual cortex, retrosplenial, sensorimotor, frontal, striatum, hippocampus, thalamus, and midbrain. We showed that the temporal structure of spike trains distinguished between low- and high-motion epochs, and again provided orthogonal information to the firing rate code. Spiking patterns become more homogeneous during high-motion epochs, which is consistent with the notion that arousal improves the reliability of signal transmission in the cortex [34].

### Outlook

Looking forward, SpikeShip opens new avenues to study temporal sequences in high-dimensional neural ensembles. Recent technological developments now allow for recordings of thousands of neurons simultaneously, either using electrical recordings or two-photon imaging [9]. The technique developed here is applicable to both kinds of data, due to linear computation time. Application of SpikeShip to such kind of data might generate important insights into the role of temporal sequences in sensory coding and memory consolidation.

## Materials and methods

### Derivation of SpikeShip

Here we derive a new dissimilarity measure, called SpikeShip, which has computational complexity O(N) for one pair of epochs. In SpikeShip, we use a cost on the absolute rather than quadratic differences in spike timing, which has two principal reasons: First, using absolute difference allows for an efficient computation of SpikeShip with computational complexity O(Nn) (number of neurons time number of spikes). Second, using the absolute instead of quadratic distance avoids over-weighing large spike time shifts (i.e. a shift from e.g. 0 to 0.1 s is weighted similarly as a shift from 1 to 1.1).

In SpikeShip, we assign a unit mass to the spike train in a given trial. For each neuron *j* in epoch *k* for which the number of spikes *n*_*k*,*j*_ > 0, we define the point process with unit energy

$$\begin{array}{c} \rho_{k,j}\left(t\right)=\frac{1}{n_{k,j}}\sum_{u=1}^{n_{k,j}}\delta \left(t-t_{k,j,u}\right)\;. \end{array}$$ (3)

This defines for each pair of neurons (*i*, *j*) in epoch *k* the cross-correlation function

$$\begin{array}{c} s_{i,j,k}\left(\tau \right)=\sum_{t=0}^{T}\;\rho_{k,i}\left(t\right)\rho_{k,j}\left(t+\tau \right)\;, \end{array}$$ (4)

Consider two epochs (*k*, *m*). We wish to find for each neuron (in epoch *k*) a transport of mass from *t* to *t*′, [**M**]_*j*,*t*,*t*′_, such that *s*_*i*,*j*,*k*_(*τ*) = *s*_*i*,*j*,*m*_(*τ*) for all (*i*, *j*, *τ*). The mass here consists of the spikes, which have a sum of 1. The objective is then to find a matrix of flows **M** that minimizes the total mover cost, i.e.

$$\begin{array}{c} \underset{M}{\text{arg min}}\sum_{j,t,t^{\prime }}\;M_{k,t,t^{\prime }}d\left(t,t^{\prime }\right)\; \end{array}$$ (5)

where *d*(*t*,*t*′) = |*t* − *t*′|.

Note that the crucial difference to a previous measure SPOTDis [27] is the following: In SPOTDis, we had a similar goal, but we computed dissimilarity via EMD for each neuron pair (*i*, *j*) separately, on the normalized cross-correlation functions *s*_*i*,*j*,*k*_(*τ*) and *s*_*i*,*j*,*m*_(*τ*) (that is, the minimum mover cost to equate one pair of cross-correlations). However, this is computationally very expensive (order *N*^2^ for *N* neurons) and only considers second-order correlations. With SpikeShip, we wish to solve the general transport problem, by shifting the spike mass for each neuron separately, such that *s*_*i*,*j*,*k*_(*τ*) = *s*_*i*,*j*,*m*_(*τ*) for all (*i*, *j*, *τ*). This, by definition, amounts to a dissimilarity measure over the entire multi-neuron spiking pattern, rather than considering all the pairwise correlations separately (as in case of SPOTDis). Yet, similar to SPOTDis, SpikeShip is exclusively based on relative spike-timing relations between neurons rather than absolute spike timing. We show that, surprisingly, the transport objective stated in Eq 5 can be achieved elegantly with order *N* neurons computational complexity, rather than order *N*^2^.

#### SpikeShip for a single spike per epoch

To derive the SpikeShip measure, we first consider the simplified case where each *i*th neuron fires one spike for all *M* epochs. Let ${\vec{f}}_{km}=\left(f_{1,km},\ldots ,f_{N,km}\right)$ be a vector of flows for each of *N* neurons in epoch *m*. In other words, *f*_1,*km*_ is the shift of the spike fired by the first neuron in the *m*th epoch. The total moving cost equals

$$\begin{array}{c} F_{km}\equiv \frac{1}{N}\sum_{i=1}^{N}\left|f_{i,km}\right|\;. \end{array}$$ (6)

The problem statement is to find a flow vector ${\vec{f}}_{km}$ such that after moving the spikes, the resulting spike train patterns are identical between epoch *k* and epoch *m*, in terms of the full matrix of spike timing relationships. We wish to find the flow vector that satisfies this constraint with minimum cost *F*_*km*_.

**Example:** Suppose there are two epochs for *N* = 6 neurons with spike times ${\vec{t}}_{k}=\left(10,10,10,10,10,10\right)$ and ${\vec{t}}_{m}=\left(20,30,35,45,50,60\right)$. We will show that the flow vector with minimum cost, such that spike patterns have identical temporal structure, equals ${\vec{f}}_{km}=\left(-20,-10,-5,5,10,20\right)$ (Fig 1).

More formally, let *t*_*m*,*i*_ be the timing of the spike for the *i*th neuron in the *m*th epoch. We denote the spike times after (post) moving them in epoch *m* as

$$\begin{array}{c} t_{m,i}^{\text{post}}\equiv t_{m,i}-f_{i} \end{array}$$ (7)

∀*i*, where we omitted the subscripts *k*, *m* from the variable *f*_*i*,*km*_ for simplicity. The constraint that all the across-neuron spike timing relationships should be identical after moving implies that

$$\begin{array}{c} t_{m,i}^{\text{post}}-t_{m,j}^{\text{post}}=t_{k,i}-t_{k,j}\;\forall i,j\in N\;. \end{array}$$ (8)

In other words, the delay between two spikes from two different neurons (*i*, *j*) should be identical between pattern *k* and *m* after moving the spikes. Substituting based on Eq 7, this can be expressed as

$$\begin{array}{c} \left(t_{m,i}-f_{i}\right)-\left(t_{m,j}-f_{j}\right)=t_{k,i}-t_{k,j}\;. \end{array}$$ (9)

Let *c*_*i*_ be the shift in spike timing for each neuron in epoch *m*, such that the spike train patterns become identical,

$$\begin{array}{c} c_{i}\equiv t_{m,i}-t_{k,i}\;. \end{array}$$ (10)

Note that with this definition of *c*_*i*_, the equation

$$\begin{array}{c} \left(t_{m,i}-c_{i}\right)-\left(t_{m,j}-c_{j}\right)=t_{k,i}-t_{k,j} \end{array}$$ (11)

holds for all (*i*, *j*). For all *i*, we can express *f*_*i*_ as a function of the shift *c*_*i*_, such that

$$\begin{array}{c} f_{i}\equiv c_{i}-g_{i}\;. \end{array}$$ (12)

We wish to solve

$$\begin{array}{c} {\vec{g}}_{\text{min}}\equiv \underset{\vec{g}}{\text{arg min}}\;F_{km}=\underset{\vec{g}}{\text{arg min}}\sum_{i}^{N}\left|c_{i}-g_{i}\right| \end{array}$$ (13)

under the constraint of Eq 9.

From Eqs 9, 11 and 12 it follows that

$$\begin{array}{c} \left(t_{m,i}-c_{i}+g_{i}\right)-\left(t_{m,j}-c_{j}+g_{j}\right)=\left(t_{m,i}-c_{i}\right)-\left(t_{m,j}-c_{j}\right) \end{array}$$ (14)

Hence the equation *g*_*i*_ = *g*_*j*_ holds for all (*i*, *j*). Thus, we can rewrite Eq 13 to

$$\begin{array}{c} g^{\text{min}}\equiv \underset{g}{\text{arg min}}\sum_{i}^{N}\left|c_{i}-g\right| \end{array}$$ (15)

to find a global shift that minimizes the L1-norm of the residuals. The solution to this equation is

$$\begin{array}{c} g^{\text{min}}=\text{Median}\left\{\vec{c}\right\}\;. \end{array}$$ (16)

Thus, our main result is that the original shifts between the two spiking patterns can be written as the decomposition

$$\begin{array}{c} c_{i}=g^{\text{min}}+f_{i}\;, \end{array}$$ (17)

for all *i*, i.e. the optimal transport in terms of neuron-specific shifts and a global temporal rigid translation term. Thus, the optimal transport between two spiking patterns (e.g. after stimulus onset) can be decomposed into the optimal transport in terms of neuron-specific shifts and a global temporal rigid translation term.

#### Global shift definition for multiple spikes

We now consider the case where in each epoch, every neuron fires a variable number of spikes. We will show that a similar derivation for SpikeShip can be made based on the weighted median. Let *n*_*i*,*k*_ and *n*_*i*,*m*_ be the number of spikes for the *i*-th neuron in epoch *k* and *m*. We will also assign a weight to each spike, such that the total weight per neuron in the computation of SpikeShip is equal. In order to do so, we first find the smallest common multiple of the spike counts for each neuron, denoted $n_{i}^{*}$. For instance, if *n*_*i*,*k*_ = 2 and *n*_*i*,*m*_ = 6 then the Least common multiple equals 6. We now replicate each spike $\frac{n_{i}^{*}}{n_{i,k}}$ and $\frac{n_{j}^{*}}{n_{i,m}}$ times. Each spike will now have a weight of $w_{i}\equiv \frac{1}{n_{i}^{*}}$. (Note that in the actual computation, we do not replicate the spikes in practice, but use an algorithm similar to the one detailed in [27]). Then, based on the optimal transport cost (EMD), we obtain shifts *c*_*i*,*u*_ for the *u*-th spike of the *i*-th neuron, $u=(1,\ldots ,n_{i}^{*})$. Note that we have shown EMD for spike trains can be efficiently computed by first shifting the mass from the most left-ward spike (i.e. first spike) out of $n_{i}^{*}$ spikes in epoch *m* to the most left-ward spike in epoch *k* [27], and then proceeding with the second spike, etc.

We use a similar derivation as the one above. Let *t*_*m*,*i*,*u*_ be the timing of the *u* − *th* replicated spike for neuron *i* in the *m*th epoch, for all (*i*, *m*, *u*), $u=(1,\ldots ,n_{i}^{*})$. We denote the spike times after (post) moving them in epoch *m* as

$$\begin{array}{c} t_{m,i,u}^{\text{post}}\equiv t_{m,i,u}-f_{i,u} \end{array}$$ (18)

∀(*i*, *u*), where we omitted the subscripts *k*, *m* from the variable *f*_*i*,*km*,*u*_ for simplicity. The constraint that all the across-neuron spike timing relationships should be identical after moving implies that

$$\begin{array}{c} t_{m,i,u}^{\text{post}}-t_{m,j,u}^{\text{post}}=t_{k,i,u}-t_{k,j,u}\; \end{array}$$ (19)

In other words, the delay between two spikes from two different neurons (*i*, *j*) should be identical between pattern *k* and *m* after moving the spikes. Substituting based on Eq 18, this can be expressed as

$$\begin{array}{c} \left(t_{m,i,u}-f_{i,u}\right)-\left(t_{m,j,u}-f_{j,u}\right)=t_{k,i,u}-t_{k,j,u}\;. \end{array}$$ (20)

There is one additional constraint, namely

$$\begin{array}{c} \left(t_{m,i,u}-f_{i,u}\right)-\left(t_{m,i,v}-f_{i,v}\right)=t_{k,i,u}-t_{k,i,v}\;. \end{array}$$ (21)

In other words, all the pairwise relationships after moving within the same neuron should be identical.

Let *c*_*i*,*u*_ be the shift in spike timing for each neuron in epoch *m*, such that the spiking patterns in the window become identical,

$$\begin{array}{c} c_{i,u}\equiv t_{m,i,u}-t_{k,i,u}\;. \end{array}$$ (22)

Note that with this definition of *c*_*i*,*u*_, the equation

$$\begin{array}{c} \left(t_{m,i,u}-c_{i,u}\right)-\left(t_{m,j,u}-c_{j,u}\right)=t_{k,i,u}-t_{k,j,u} \end{array}$$ (23)

holds for all (*i*, *j*, *u*). For all *i*, *u*, we can express *f*_*i*,*u*_ as a function of the shift *c*_*i*,*u*_, such that

$$\begin{array}{c} f_{i,u}\equiv c_{i,u}-g_{i,u}\;. \end{array}$$ (24)

Define $\vec{g}\equiv ((g_{i,1},\ldots ,g_{i,n_{i}^{*}}),\ldots ,(g_{N,1},\ldots ,g_{N,n_{N}^{*}}))$.

We wish to solve

$$\begin{array}{c} {\vec{g}}_{\text{min}}\equiv \underset{\vec{g}}{\text{arg min}}\sum_{i}^{N}\sum_{u}^{n_{i}^{*}}w_{i,u}\left|c_{i,u}-g_{i,u}\right| \end{array}$$ (25)

under the constraints of Eqs 20, 21 and 23.

Given these two constraints, the equation *g*_*i*,*u*_ = *g*_*j*,*u*_ holds for all (*i*, *j*, *u*). Thus, we can rewrite Eq 25 to

$$\begin{array}{c} g^{\text{min}}\equiv \underset{g}{\text{arg min}}\sum_{i}^{N}\sum_{u}^{n_{i}^{*}}w_{i,u}\left|c_{i,u}-g\right|\; \end{array}$$ (26)

to find a global translation term that minimizes the L1-norm of the residuals. The solution to this equation is the weighted median *g*,

$$\begin{array}{c} g^{\text{min}}=\text{WeightedMedian}((c_{1,1},w_{i,u},\ldots ,c_{1,n_{1}^{*}},w_{1,n_{1}^{*}}),\ldots ,(c_{N,1},w_{N,1},\ldots ,c_{N,n_{N}^{*}},w_{N,n_{N}^{*}}))\;. \end{array}$$

Now average mover cost, *SpikeShip*, equals

$$\begin{array}{c} F_{km}\equiv \frac{1}{\left|A_{km}\right|}\sum_{i\in A_{km}}\frac{1}{n_{i}^{*}}\sum_{u}^{n_{i}^{*}}\left|f_{i,u}\right|\;. \end{array}$$ (27)

where $A_{km}\equiv \left\{i:n_{i,m}>0\wedge n_{i,k}>0\right\}$ is the set of neurons that are active in both epochs *k* and *m*. Thus, we derive the result that the original shifts between the two spiking patterns can be written as the decomposition

$$\begin{array}{c} c_{i,u}=g^{\text{min}}+f_{i,u}\;, \end{array}$$ (28)

for all (*i*, *u*), i.e. the optimal transport in terms of neuron-specific shifts and a global translation term. Thus, the optimal transport between two spiking patterns (e.g. after stimulus onset) can be decomposed into the optimal transport in terms of neuron-specific shifts and a global temporal rigid translation term. This computation has linear complexity O(N). We base the computation of the weighted median on [47, 48] and an adaptation of the *Robustats* [49] Python library. In practice, the computation of *c*_*i*,*u*_ (optimal transport per neuron) can be efficiently performed not by replicating the spikes to the common multiple integer (used in the derivation above), but by using a similar algorithm as in [27].

### Comparison with other spike train metrics

#### Victor-Purpura distance

Victor-Purpura metric (VP) combines both rate and temporal information by defining a hyper-parameter *q* related with the cost of shift between spikes. Thus, VP extracts rate information for small values of *q*, converging to the absolute difference of spike counts for two spike trains with *n*_*i*_ and *n*_*j*_ spikes when *q* = 0 (i.e., *VP*(*q* = 0) = |*n*_*i*_ − *n*_*j*_|). On the other hand, for high values of *q*, VP distance maximizes the contribution of timing coding, converging to the sum of the total spike of both spike trains (i.e., *VP*(*q* → ∞) = *n*_*i*_ + *n*_*j*_)). We used *Elephant* (Electrophysiology Analysis Toolkit) [50] to compute VP distance.

Finally, we note that there is some relation between VP distance and EMD (Earth Mover Distance), as VP also contains a transport term (in the sense of shifting spikes). In case that two spike trains have an identical value of *n*, the VP becomes equal to the *n*/*T* ⋅ *EMD* as *q* → 1/*T* from below. In this case VP will ignore the insertion costs and only contain shift cost (as the maximum shift cost equals *Tq* → 1). We have added some simulations to show indeed that VP and the scaled (by *N*/*T*) EMD are identical in this case of equal spike counts. However, if the spike count is different between two spike trains, then VP and EMD will diverge. We furthermore note that the crux of SpikeShip is of course to consider inter-neuronal timing relationships, which is achieved by decomposing the total moving cost into a global flow and a neuron-specific flow costs. This means that VP and SpikeShip remain fundamentally different measures. We have illustrated this results in S16 Fig.

#### SPIKE and RI-SPIKE distances

Both SPIKE and RI-SPIKE measure the similarity of two spike trains in terms of the absolute spike timing. RI-SPIKE was developed to avoid a rate bias in the computation of the dissimilarity. To perform the analyses for SPIKE and RI-SPIKE metrics, we used a python library for spike train similarity analysis called *PySpike* [51].

#### SPOTDis

Previously, we have developed a dissimilarity measure between multi-neuron temporal spiking patterns called SPOTDis (Spike Pattern Optimal Transport Dissimilarity) [27]. SPOTDis is defined as the minimum energy (optimal transport) that is needed to transform all pairwise cross-correlations of one epoch *k* into the pairwise cross-correlations of another epoch *m*. This optimal transport is given by EMD. SPOTDis only measures pairwise correlations and has computational cost of order *N*^2^. We used SPOTDis python module [27] to perform the analyses.

### Application in high-dimensional neural data

#### Allen Brain Institute datasets

We used the free, publicly available datasets of Allen Brain Institute through AllenSDK (For more details, see http://help.brain-map.org/display/observatory/Documentation). Neuropixels silicon probes [9] were used to record neurons with precise spatial and temporal resolution [12].

We selected the cells of 32 mice during natural scene presentations. The cells were selected considering a signal-noise ratio (*SNR*) such that *SNR* > 0. The neural activity from a total of *N* = 8, 301 cells was selected from the Primary visual area (VISp), Lateral visual area (VISl), Anterolateral visual area (VISal), Posteromedial visual area (VISpm), Rostrolateral visual area (VISrl), and Anteromedial visual area (VISam).

The computation time for SpikeShip to compute one dissimilarity matrix of Fig 4C was on average 58.8 secs (see Methods), thus indicating a highly effective computation time for *N* = 8, 301 neurons. In contrast, we estimate the computation time for the previous SPOTDis measure [27] to be approximately 175.71 hours (1 week and 7.7 hours; based on *n* ≈ 3.33 spikes per neuron).

#### Spontaneous activity

For the spontaneous activity dataset, we used free, publicly available datasets [22]. It contains data from three mice: “Krebs” (*N* = 1, 462), “Robbins”(*N* = 2, 296), and “Waksman” (*N* = 2, 688). Probes were located in distinct cortical areas (visual, sensorimotor, frontal, and retrosplenial), hippocampus, thalamus, striatum, and midbrain [10]. In this study, Singular Value Decomposition (SVD) was applied to the recordings of the pixel difference between consecutive frames of a mouse face movie. Thus, each experiment contains information about the recorded spike times, cells, and the processed SVD components of whisker’s motion.

For our analyses, we sum the first four SVD components (Fig 6A) and then we subtracted the median across dimensions (denoising, see Fig 6B). A sliding window was used to sum the whisker’s motion across the interval [*t*, *t* + 0.5] second (Fig 6C and 6D). We used Hidden Markov Model (HMM) to automatically detect states of low- and middle- & high-motion epochs. Then, we selected one hundred epochs for both low- and high-motion states based on the SVD motion value, and ran SpikeShip across different amounts of cells, depending on the experiment. We used the implementations of HMM, t-SNE, and Spectral Embedding from the python library Scikit Learn v0.22.1.

For these experiments, SpikeShip took 25.5 secs in creating a dissimilarity matrix. Considering *N* ≈ 2, 500, *n* ≈ 5, based on Fig 6D, the computation time of SPOTDis can be estimated as 34.06 hours (1 day and 10.06 hours; *SU* ≈ 4, 808).

#### Computation of Euclidean distance between two firing rate vectors

For a population of *N* neurons, we computed the firing rate vectors ($\vec{FR}$) for each epoch of our analyses as the count of spikes per neuron divided by a window length *T*. For example, for two neurons with spikes *N*_0_ = (0.0, 0.1, 0.5) and *N*_1_ = (0.0, 0.2, 0.4, 1.0) and *T* = 1, the firing rate vectors correspond to $\vec{FR}=\left(\frac{|N_{0}|}{T},\frac{|N_{1}|}{T}\right)=\left(3,4\right)$.

Additionally, we normalized the firing rate vectors across epochs (z-score). For example, for two epochs *k* and *m* and normalized firing rate vectors of $\vec{FR_{k}}=\left(3,4,5\right)$ and $\vec{FR_{m}}=\left(2,5,3\right)$, mean and standard deviation of firing rates across epochs correspond to $\vec{\mu }=\left(2.5,4.5,4\right)$ and $\vec{\sigma }=\left(0.5,0.5,1\right)$, respectively. Then, z-scored firing rate vectors are $\vec{FR_{k}^{*}}=\left(\frac{3-2.5}{0.5},\frac{4-4.5}{0.5},\frac{5-4}{1}\right)=\left(1,-1,1\right)$ and $\vec{FR_{m}^{*}}=\left(\frac{2-2.5}{0.5},\frac{5-4.5}{0.5},\frac{3-4}{1}\right)=\left(-1,1,-1\right)$.

Finally, we computed the Euclidean distance between both normalized vectors is $d_{Euc}=\sqrt{\sum_{i}^{N}{\left(\vec{FR_{m,i}^{*}}-\vec{FR_{k,i}^{*}}\right)}^{2}}=\sqrt{12}\approx 3.46$.

#### Discriminability index

Since the information in temporal spiking sequences compared to the information from traditional firing rate codes could differ, we wanted to quantify to what extent the degree of discriminability within and between different visual stimuli differ in a dissimilarity matrix *diss* (i.e. within and between natural images) per stimulus *id*.

To this end, we computed a “Discriminability index” (*d*), defined as the difference between the average distance within and between stimulus *id*, divided by the squared sum of their variances to the power of two.

$$\begin{array}{c} d_{diss,id}=\frac{\mu_{diss,between,id}-\mu_{diss,within,id}}{\sqrt{s_{diss,between}^{2}+s_{diss,within}^{2}}} \end{array}$$ (29)

Therefore, this index indicates how many standard deviations are two sets of distances away from each other.

## Supporting information

S1 FigIllustration of SpikeShip for multiple spikes.(Top) Example of two epochs with spike times for two active neurons *N*_0_ and *N*_1_ (i.e., *A*_*km*_ = 2): *t*_*k*_ = ((10, 15), (10)) and *t*_*m*_ = ((35, 40, 45), (35, 40)). Spike counts per epoch *m* and *k* correspond to (|*t*_*N*1,*k*_| = 2, |*t*_*N*1,*m*_| = 1) and (|*t*_*N*0,*k*_| = 3, |*t*_*N*1,*k*_| = 2), respectively. (Middle) The difference of spike times is computed by normalizing the mass across neurons and between epochs. Such spike time difference is $\vec{c}=\left(15,10,30,30,25,30,25,25\right)$ with mass (i.e., weights) $W=(\frac{1}{2},\frac{1}{2},\frac{1}{6},\frac{1}{6},\frac{1}{6},\frac{1}{6},\frac{1}{6},\frac{1}{6})$. Then, the global shift (i.e., *g*^*min*^) equals 20. (Bottom) Neuron-specific shifts correspond to $\vec{f}=W\cdot \left(\vec{c}-\vec{g}\right)=\left(\frac{-5}{2},\frac{-10}{2},\frac{10}{6},\frac{10}{6},\frac{5}{6},\frac{10}{6},\frac{5}{6},\frac{5}{6}\right)$. Thus, $F_{km}=\frac{1}{A_{km}}\sum_{i\in A_{km}}\frac{1}{n_{i}^{*}}\sum_{u}^{n_{i}^{*}}\left|f_{i,u}\right|=\frac{15}{2}=7.5$.(PDF)

S2 Fig Accuracy and Speed-up comparison for single- and multi-spike patterns. A) Example of single spike trains for two epochs for 10 neurons. Patterns were generated as uniform sequences with *n* = 1 spike per neuron per epoch. B) Computational speed-up (log-scale) for SpikeShip vs. SPOTDis (serial execution) for increasing amount of neurons *N*. Speed-up is approximately *N* when there is 1 spike per neuron, and it increases when *n* > 1 (i.e. the multi-spike pattern case). C) Example of three single-spike patterns: (−20, 0, 0, +20), (0, 0, 0, 0), and (−15, −15, +15, +15), from left to right. SpikeShip assigns a geometrically more appropriate transport cost between pattern 1 and 2 (*F*_1,2_ = 10) than SPOTDis (*D*_1,2_ = 12.5), considering their distance with pattern 0.
(PDF)

S3 Fig Multimodal activation and deactivation patterns can be detected using SpikeShip. (A) Multiple bimodal activation patterns and examples of realizations for each pattern (*N* = 50 neurons). Simulation parameters were pulse rate λ_*in*_ = 0.35 spks/sample, baseline rate λ_*out*_ = 0.05 spks/sample, epoch window length *T*_*epoch*_ = 300 samples and pulse length *T*_*pulse*_ = 20 samples. Bottom figures show sorted dissimilarity matrix and t-SNE for simulation with patterned noise (left) and homogeneous noise (right). (B) Multiple bimodal activation patterns and examples of realizations for each pattern (*N* = 50 neurons). Simulation parameters were λ_*out*_ = 0.02 spks/sample (i.e. the deactivation period), λ_*in*_ = 0.3 spks/sample, *T*_*epoch*_ = 300 and *T*_*deactivation*_ = 150 samples. Bottom figures show sorted dissimilarity matrix and t-SNE for simulation with patterned noise (left) and homogeneous noise (right).
(PDF)

S4 Fig Performance of SpikeShip depends on the SNR but it outperforms SPOTDis. (A) Performance of clustering on 2D t-SNE embeddings for SPOTDis (left) and SpikeShip (right) measured with ARI score. Clusters detection was performed using K-Means algorithm. Firing rate inside pulse period is varied, while firing rate outside pulse was varied: λ_*in*_ and λ_*out*_ correspond to the pulse rate and the baseline rate, respectively. Thus, Pulse rate / Baseline rate corresponds to the signal-to-noise ratio (SNR). Additionally, *T*_*pulse*_ correponds to the period of time during the pulse rate and *T*_*epoch*_ to the window length. We simulated 5 patterns with 30 repetitions each, with λ_*out*_ = 0.05 spks/sample, and λ_*in*_ attaining values of 0.15, 0.2, 0.25, 0.35, 0.45 or 0.5 spks/sample, *T*_*pulse*_ = 30 and *T*_*epoch*_ = 1000 samples. The number of neurons was 25, 50 or 100, and 150 epochs of homogeneous noise. We show the mean and the standard deviation across 10 repetitions of the same simulation. Performance relative to ground truth increases with SNR. Lower SNRs are needed for achieving the same performance when the number of neurons is larger. (B) as (A), but now varying the pulse duration. Simulation parameters were λ_*out*_ = 0.05 spks/sample, and λ_*in*_ = 0.5, 0.4, 0.3, 0.2, 0.1 spks/sample, and *T*_*pulse*_ of 100, 200, 300, 400 or 500 samples, with *T*_*epoch*_ = 1000 samples; note that the product of λ_*in*_. *T*_*pulse*_ remained constant.
(PDF)

S5 Fig Dependence of clustering performance on chosen window length and temporal jitter of spike pattern onset. (A) Each pattern has a length of 300 samples, and is embedded in a larger window starting from -300 samples to +300 samples, with homogeneous noise surrounding the pattern on the left and right. The onset of the pattern is -150 samples plus some random offset Δ*t*_*w*_. For each epoch realization, the value of Δ*t*_*w*_ was randomly chosen with uniform probability from an interval determined by the maximum window offset (max offset of 100 meant that Δ*t*_*w*_ ∈ [-100,100]). We select a window ranging from −*T*_*w*_/2 to +*T*_*w*_/2 samples of length *T*_*w*_. (B) Clustering performance of 2D t-SNE embeddings was measured relative to ground-truth (ARI, compared with k-Means labels) and with an unsupervised performance measure, Silhouette Score. Clustering performance decreased as the maximum window offset increased, due to the inclusion of noise spikes around the spike pattern. SpikeShip has a small but consistent performance advantage relative to SPOTDis. Furthermore, SpikeShip strongly outperformed VP results in clustering performance, which as expected was severely distorted by global shifts in spiking patterns. ARI and Silhouette scores correspond to the mean value obtained across 10 repetitions for each combination of window length (*T*_*w*_) and max window offset (Δ*t*_*w*_).
(PDF)

S6 Fig Comparison of VP and SpikeShip for simulations with multiple patterns and global shifts. We simulated 6 patterns with Poisson noise surrounding the pattern on the left and right. The onset of the pattern (*i*.*e*., Δ*t*_*w*_) was randomly assigned between 0 and 0.8 with a window length of *T*_*w*_ = 0.2*s* for the patterns. The analysis window used here is 1 s, i.e. the entire period. SpikeShip correctly detects the 6 different patterns, but also SpikeShip can decompose the spike patterns to make it invariant to changes in global shifts. VP distance drastically depends on the global shift applied to the spikes. SpikeShip can retrieve the global shift from the spike sequences and reconstruct the random global shifts applied to the spike trains.
(PDF)

S7 Fig Performance of VP distance is affected by changes in both local and global scaling rates. A) Global scaling. Same simulations as in S8 Fig. Victor-Purpura distance (VP) was used with different values of *q*. When *q* = 0, *VP* = |*n*_*i*_ − *n*_*j*_|, with *n*_*i*_ and *n*_*j*_ the spike count of spike sequences *i* and *j*, respectively. Epochs are clustered based on rates. B) Local scaling. Same simulations as in S9 Fig. VP distance was used with different values of *q*. When *q* → ∞, *VP* = *n*_*i*_ + *n*_*j*_. Besides high values of *q* aim to extract temporal information from spike trains, these 2D embeddings demonstrate that the contribution between rate and timing using VP is difficult to interpret and very sensitive to noise.
(PDF)

S8 Fig Performance of SpikeShip is not affected by a global scaling rate. Two different temporal patterns with different firing rates. Each temporal pattern can occur in a low (λ_*in*_ = 0.2 and λ_*out*_ = 0.02 spks/sample), medium (λ_*in*_ = 0.4 and λ_*out*_ = 0.04 spks/sample) or high rate (λ_*in*_ = 0.7 and λ_*out*_ 0.07 spks/sample) state, with a constant ratio of λ_*in*_/λ_*out*_. In addition, the noise pattern can also occur in one of three rate states. The pulse duration was 30 samples. Shown at the bottom the sorted dissimilarity matrix with SpikeShip values, the t-SNE embedding with the ground-truth cluster labels and the t-SNE embedding with the HDBSCAN cluster labels.
(PDF)

S9 Fig Performance of SpikeShip is not affected by a local scaling rate. Two different temporal patterns with different firing rates. Each temporal pattern could occur in one of two rates states: In the first rate state, the first 25 neurons are firing at a low rate (λ_*in*_ = 0.3 and λ_*out*_ = 0.03 spks/sample), and the other 25 are firing at a high rate (λ_*in*_ = 0.7 and λ_*out*_ = 0.07 spks/sample). In the second rate state, the rate scaling is reversed. The pulse duration was 30 samples. Shown at the bottom the sorted dissimilarity matrix with SpikeShip values, the t-SNE embedding with the ground-truth cluster labels and the t-SNE embedding with the HDBSCAN cluster labels.
(PDF)

S10 Fig Performance of SPIKE and RI-SPIKE are affected by both global and local scaling. A) Global scaling. SPIKE and RI-SPIKE computations for globally scaled sequences. Top: dissimilarity matrices sorted by pattern id and scaling factor. Bottom: 2D t-SNE embeddings of epochs. B) Local scaling. SPIKE and RI-SPIKE computations for locally scaled sequences. Both A and B were computed using the same simulations as in S8 Fig. Top: dissimilarity matrices sorted by pattern id and scaling factor. Bottom: 2D t-SNE embeddings of epochs. The first 180 epochs correspond to noise.
(PDF)

S11 Fig Comparison of spike train metrics across single sessions during Natural Scene presentations. A) Pairwise comparison of epochs and clustering performance of metrics (one session). Top: Dissimilarity matrices sorted by Natural scene ID. Bottom: 2D t-SNE embeddings. Clustering performance was computed via Adjusted Rank Index (*ARI*). We used Gaussian Mixture (GM) to assign labels to each cluster. B) Distribution of clustering performances (*ARI*_*GM*_) across sessions. For VP distances, the average number of spike times (*n*) was computed for every session. The mean and standard deviation of *n* and *N* across all the sessions were (*μ*_*n*_ = 2.1, *σ*_*n*_ = 0.26) and (*μ*_*N*_ = 570.4, *σ*_*N*_ = 190.6), respectively. Median values of *ARI*_*GM*_ for Firing rates, RI-SPIKE, and SpikeShip are 0.1848, 0.075, and 0.1865, respectively. We performed the Welch’s t-test across epochs. The obtained *p*-values from comparison of SpikeShip and all the other metrics were: (Firing Rates, SpikeShip) = 6.71 × 10^−2^, (RI-SPIKE, SpikeShip) = 5.49 × 10^−9^.
(PDF)

S12 Fig Comparison between clusters from SpikeShip and Firing rates embeddings of Natural scenes. A) 2D t-SNE embeddings from SpikeShip and Firing rates’ dissimilarity matrices. The allocation of natural scenes’ clusters are different between the two embeddings. Natural scenes are represented by their ID. B) Scaled Euclidean pairwise distance between centroids of each cluster for both SpikeShip (Left) and firing rates (Middle), and their difference (Right).
(PDF)

S13 Fig Analysis of large scale neural recordings during visual stimuli presentations with SPIKE and RI-SPIKE. Top: Dissimilarity matrices sorted by Natural Scene ID. Middle: 2D t-SNE embeddings from dissimilarity matrices colored by Natural Scene ID. Bottom: The clustering performance through ARI score and Spearman correlation between dissimilarity matrices computed via SPIKE, RI-SPIKE, Firing rates, and SpikeShip. The clustering performance of SPIKE and RI-SPIKE is lower than the clustering performance by using the traditional firing rates and SpikeShip. SPIKE and RI-SPIKE are highly correlated while Firing Rates and SpikeShip are highly uncorrelated.
(PDF)

S14 Fig Spontaneous activity analyses for 3 mice. Multi-spike sequence analyses for three mice (rows). Left: dissimilarity matrices. Middle: 2D t-SNE embedding. Right: 2D Spectral Embedding (Laplacian Eigenmaps).
(PDF)

S15 Fig Variability of epochs allows distinction between low-motion and middle- & high-motion epochs. Normalized distribution of L2-distances between each epoch to the centroid for t-SNE and Spectral embedding (SE). We performed the Welch’s t-test between low-motion and middle- & high-motion epochs (variances between two groups were smaller than 5 × 10^−4^). Thus, (*) if *p*-*value* < 0.005, (**) if *p*-*value* < 0.05, and (***) if *p*-*value* < 0.5 (i.e., evidence against null hypothesis of equal population means).
(PDF)

S16 Fig Relation between VP and EMD distances for spike trains with same spike counts. Simulations of spike trains with same number of spikes (i.e., *n* = 20). Top: Comparison of distances between VP and EMD. Spike trains were defined as *t*_*m*_ = (0, …, 0) and *t*_*k*_ = (*T*, …, *T*), where *T* is the window length. Left: VP(*q* = 1/*n*) reaches a maximum value until *T* = 2*n* = 40. After that threshold, VP(*q* = 1/*n*) assign insertion costs rather than shifts. Right: (*T*/*n*) VP(*q* = 1/*T*) equals EMD. Bottom: Comparison of the average distances between VP and EMD across 100 simulations. Spike times for *t*_*m*_ and *t*_*k*_ (with same spike count) were randomly generated from a uniform distribution *U*(0, *T*).
(PDF)
