# Supplementary material for: SpikeShip: A method for fast, unsupervised discovery of high-dimensional neural spiking patterns
Source: PLoS Comput Biol. 2023 Jul 31;19(7):e1011335. doi: 10.1371/journal.pcbi.1011335 (PMC10414626; doi:10.1371/journal.pcbi.1011335)
Supplement: S1 Fig — (Top) Example of two epochs with spike times for two active neurons N0 and N1 (i.e., Akm = 2): tk = ((10, 15), (10)) and tm = ((35, 40, 45), (35, 40)). Spike counts per epoch m and k correspond to (|tN1,k| = 2, |tN1,m| = 1) and (|tN0,k| = 3, |tN1,k| = 2), respectively. (Middle) The difference of spike times is computed by normalizing the mass across neurons and between epochs. Such spike time difference is c→=(15,10,30,30,25,30,25,25) with mass (i.e., weights) W=(12,12,16,16,16,16,16,16). Then, the global shift (i.e., gmin) equals 20. (Bottom) Neuron-specific shifts correspond to f→=W·(c→-g→)=(-52,-102,106,106,56,106,56,56). Thus, Fkm=1Akm∑i∈Akm1ni*∑uni*|fi,u|=152=7.5. (PDF) [file pcbi.1011335.s001.pdf]

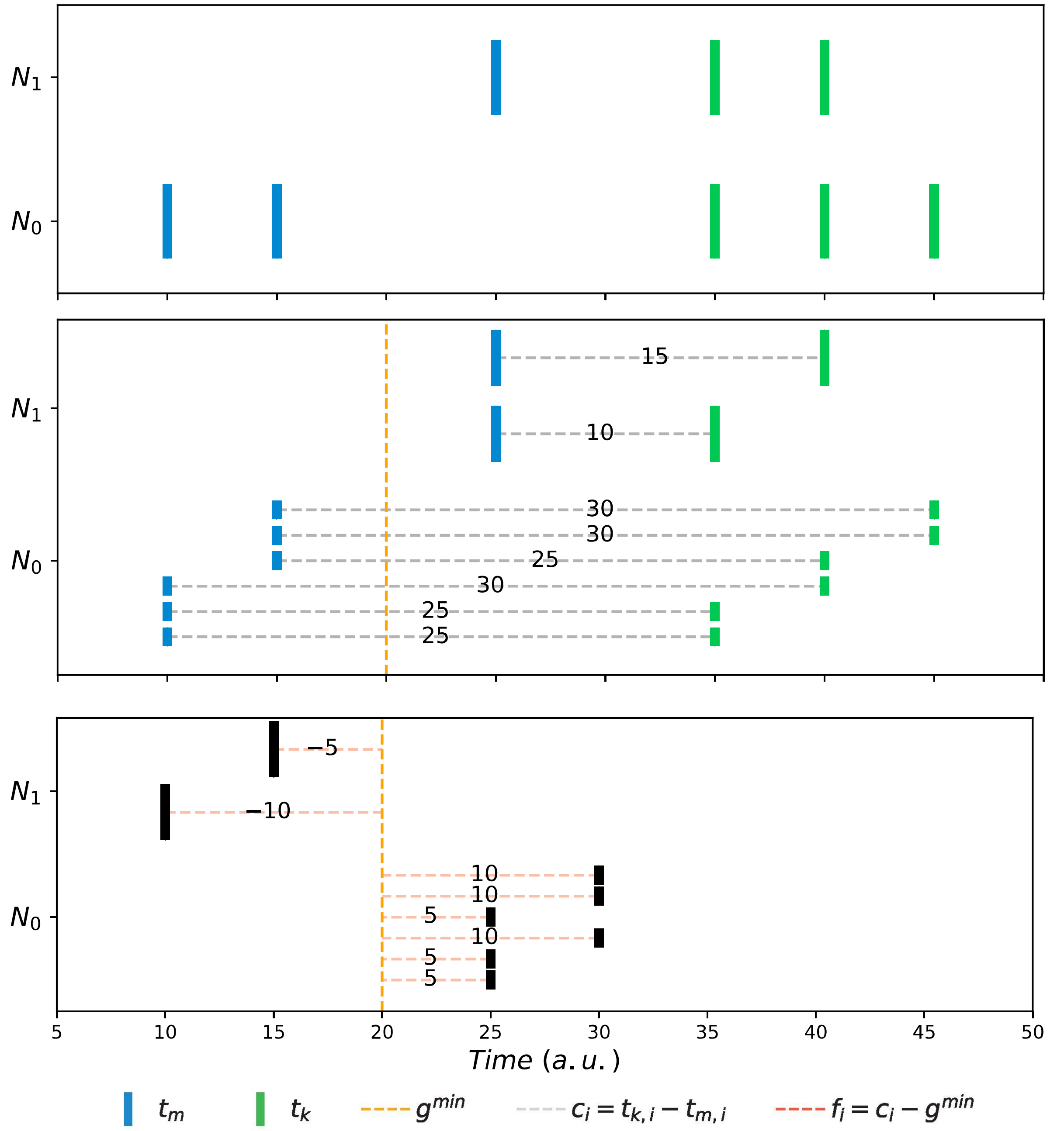

Fig S1: Illustration of SpikeShip for multiple spikes. (Top) Example of two epochs with spike times for two active neurons N_0 and N_1 (i.e., $A_{km} = 2$): $t_k = ((10, 15), (10))$ and $t_m = ((35, 40, 45), (35, 40))$. Spike counts per epoch m and k correspond to ($|t_{N_0,m}| = 2$, $|t_{N_1,m}| = 1$) and ($|t_{N_0,k}| = 3$, $|t_{N_1,k}| = 2$), respectively. (Middle) The difference of spike times is computed by normalizing the mass across neurons and between epochs. Such spike time difference is $\vec{c} = (15, 10, 30, 30, 25, 30, 25, 25)$ with mass (i.e., weights) $W = (\frac{1}{2}, \frac{1}{2}, \frac{1}{6}, \frac{1}{6}, \frac{1}{6}, \frac{1}{6}, \frac{1}{6}, \frac{1}{6})$. Then, the global shift (i.e., g^{min}) equals 20. (Bottom) Neuron-specific shifts correspond to $\vec{f} = W \cdot (\vec{c} - \vec{g}) = (\frac{-5}{2}, \frac{-10}{2}, \frac{10}{6}, \frac{10}{6}, \frac{5}{6}, \frac{10}{6}, \frac{5}{6}, \frac{5}{6})$. Thus, $F_{km} = \frac{1}{A_{km}} \sum_{i \in A_{km}} \frac{1}{n_i^*} \sum_u^{n_i^*} |f_{i,u}| = \frac{15}{2} = 7.5$.
