# Supplementary material for: SpikeShip: A method for fast, unsupervised discovery of high-dimensional neural spiking patterns
Source: PLoS Comput Biol. 2023 Jul 31;19(7):e1011335. doi: 10.1371/journal.pcbi.1011335 (PMC10414626; doi:10.1371/journal.pcbi.1011335)
Supplement: S2 Fig — A) Example of single spike trains for two epochs for 10 neurons. Patterns were generated as uniform sequences with n = 1 spike per neuron per epoch. B) Computational speed-up (log-scale) for SpikeShip vs. SPOTDis (serial execution) for increasing amount of neurons N. Speed-up is approximately N when there is 1 spike per neuron, and it increases when n > 1 (i.e. the multi-spike pattern case). C) Example of three single-spike patterns: (−20, 0, 0, +20), (0, 0, 0, 0), and (−15, −15, +15, +15), from left to right. SpikeShip assigns a geometrically more appropriate transport cost between pattern 1 and 2 (F1,2 = 10) than SPOTDis (D1,2 = 12.5), considering their distance with pattern 0. (PDF) [file pcbi.1011335.s002.pdf]

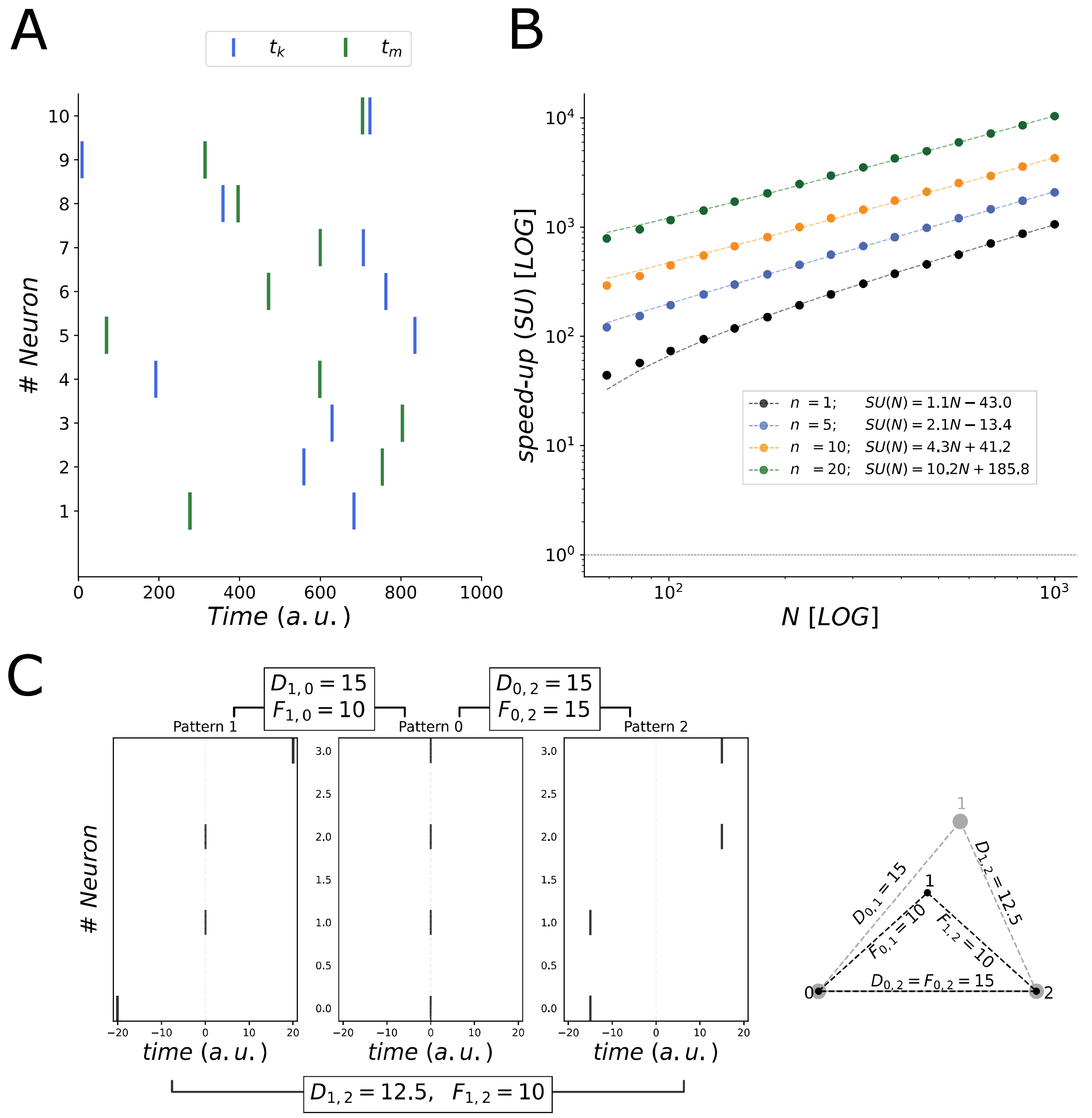

Fig S2: Accuracy and Speed-up comparison for single- and multi-spike patterns. A) Example of single spike trains for two epochs for 10 neurons. Patterns were generated as uniform sequences with $n = 1$ spike per neuron per epoch. B) Computational speed-up (log-scale) for SpikeShip vs. SPOTDis (serial execution) for increasing amount of neurons N. Speed-up is approximately N when there is 1 spike per neuron, and it increases when $n > 1$ (i.e. the multi-spike pattern case). C) Example of three single-spike patterns: $(-20, 0, 0, +20)$, $(0, 0, 0, 0)$, and $(-15, -15, +15, +15)$, from left to right. SpikeShip assigns a geometrically more appropriate transport cost between pattern 1 and 2 ($F_{1,2} = 10$) than SPOTDis ($D_{1,2} = 12.5$), considering their distance with pattern 0.
