# Supplementary material for: SpikeShip: A method for fast, unsupervised discovery of high-dimensional neural spiking patterns
Source: PLoS Comput Biol. 2023 Jul 31;19(7):e1011335. doi: 10.1371/journal.pcbi.1011335 (PMC10414626; doi:10.1371/journal.pcbi.1011335)
Supplement: S3 Fig — (A) Multiple bimodal activation patterns and examples of realizations for each pattern (N = 50 neurons). Simulation parameters were pulse rate λin = 0.35 spks/sample, baseline rate λout = 0.05 spks/sample, epoch window length Tepoch = 300 samples and pulse length Tpulse = 20 samples. Bottom figures show sorted dissimilarity matrix and t-SNE for simulation with patterned noise (left) and homogeneous noise (right). (B) Multiple bimodal activation patterns and examples of realizations for each pattern (N = 50 neurons). Simulation parameters were λout = 0.02 spks/sample (i.e. the deactivation period), λin = 0.3 spks/sample, Tepoch = 300 and Tdeactivation = 150 samples. Bottom figures show sorted dissimilarity matrix and t-SNE for simulation with patterned noise (left) and homogeneous noise (right). (PDF) [file pcbi.1011335.s003.pdf]

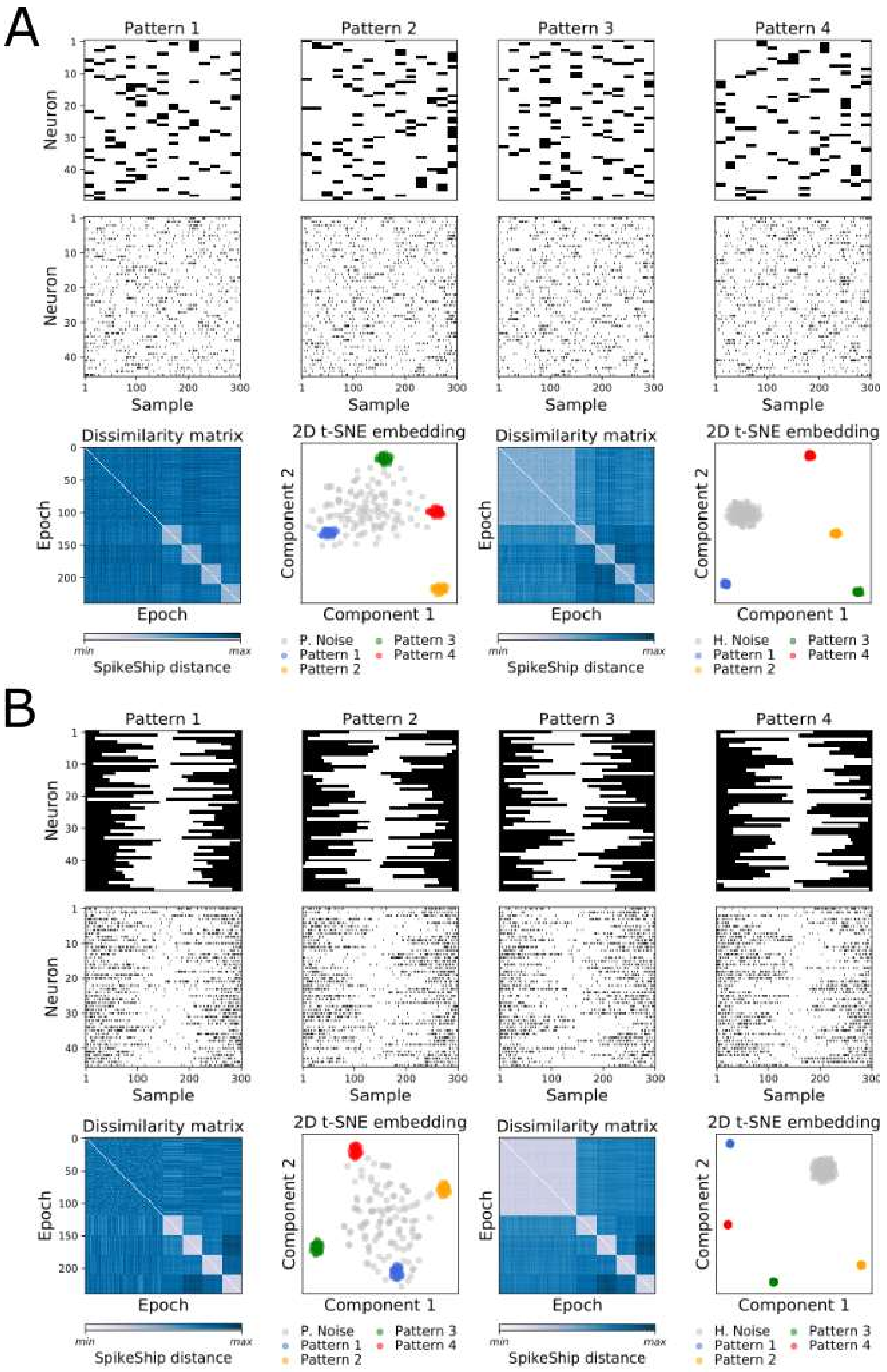

Fig S3: Multimodal activation and deactivation patterns can be detected using SpikeShip. (A) Multiple bimodal activation patterns and examples of realizations for each pattern ($N = 50$ neurons). Simulation parameters were pulse rate $\lambda_{in} = 0.35$ spks/sample, baseline rate $\lambda_{out} = 0.05$ spks/sample, epoch window length $T_{epoch} = 300$ samples and pulse length $T_{pulse} = 20$ samples. Bottom figures show sorted dissimilarity matrix and t-SNE for simulation with patterned noise (left) and homogeneous noise (right). (B) Multiple bimodal activation patterns and examples of realizations for each pattern ($N = 50$ neurons). Simulation parameters were $\lambda_{out} = 0.02$ spks/sample (i.e. the deactivation period), $\lambda_{in} = 0.3$ spks/sample, $T_{epoch} = 300$ and $T_{deactivation} = 150$ samples. Bottom figures show sorted dissimilarity matrix and t-SNE for simulation with patterned noise (left) and homogeneous noise (right).
