# Supplementary material for: SpikeShip: A method for fast, unsupervised discovery of high-dimensional neural spiking patterns
Source: PLoS Comput Biol. 2023 Jul 31;19(7):e1011335. doi: 10.1371/journal.pcbi.1011335 (PMC10414626; doi:10.1371/journal.pcbi.1011335)
Supplement: S4 Fig — (A) Performance of clustering on 2D t-SNE embeddings for SPOTDis (left) and SpikeShip (right) measured with ARI score. Clusters detection was performed using K-Means algorithm. Firing rate inside pulse period is varied, while firing rate outside pulse was varied: λin and λout correspond to the pulse rate and the baseline rate, respectively. Thus, Pulse rate / Baseline rate corresponds to the signal-to-noise ratio (SNR). Additionally, Tpulse correponds to the period of time during the pulse rate and Tepoch to the window length. We simulated 5 patterns with 30 repetitions each, with λout = 0.05 spks/sample, and λin attaining values of 0.15, 0.2, 0.25, 0.35, 0.45 or 0.5 spks/sample, Tpulse = 30 and Tepoch = 1000 samples. The number of neurons was 25, 50 or 100, and 150 epochs of homogeneous noise. We show the mean and the standard deviation across 10 repetitions of the same simulation. Performance relative to ground truth increases with SNR. Lower SNRs are needed for achieving the same performance when the number of neurons is larger. (B) as (A), but now varying the pulse duration. Simulation parameters were λout = 0.05 spks/sample, and λin = 0.5, 0.4, 0.3, 0.2, 0.1 spks/sample, and Tpulse of 100, 200, 300, 400 or 500 samples, with Tepoch = 1000 samples; note that the product of λin. Tpulse remained constant. (PDF) [file pcbi.1011335.s004.pdf]

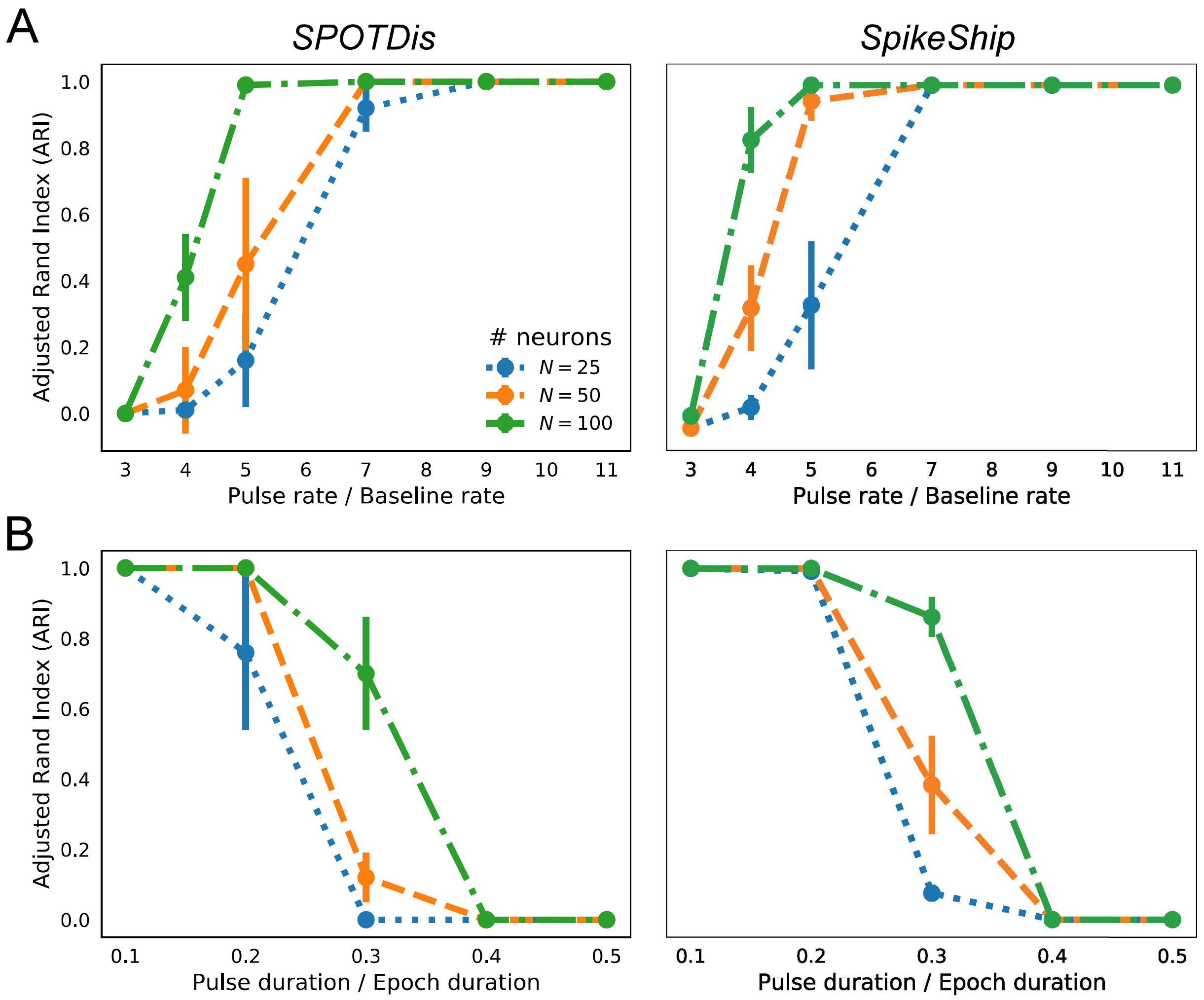

Fig S4: Performance of SpikeShip depends on the SNR but it outperforms SPOTDis. (A) Performance of clustering on 2D t-SNE embeddings for SPOTDis (left) and SpikeShip (right) measured with ARI score. Clusters detection was performed using K-Means algorithm. Firing rate inside pulse period is varied, while firing rate outside pulse was varied: λ_{in} and λ_{out} correspond to the pulse rate and the baseline rate, respectively. Thus, Pulse rate / Baseline rate corresponds to the signal-to-noise ratio (SNR). Additionally, T_{pulse} correponds to the period of time during the pulse rate and T_{epoch} to the window length. We simulated 5 patterns with 30 repetitions each, with $\lambda_{out} = 0.05$ spks/sample, and λ_{in} attaining values of 0.15, 0.2, 0.25, 0.35, 0.45 or 0.5 spks/sample, $T_{pulse} = 30$ and $T_{epoch} = 1000$ samples. The number of neurons was 25, 50 or 100, and 150 epochs of homogeneous noise. We show the mean and the standard deviation across 10 repetitions of the same simulation. Performance relative to ground truth increases with SNR. Lower SNRs are needed for achieving the same performance when the number of neurons is larger. (B) as (A), but now varying the pulse duration. Simulation parameters were $\lambda_{out} = 0.05$ spks/sample, and $\lambda_{in} = 0.5$, 0.4, 0.3, 0.2, 0.1 spks/sample, and T_{pulse} of 100, 200, 300, 400 or 500 samples, with $T_{epoch} = 1000$ samples; note that the product of λ_{in}. T_{pulse} remained constant.
