# Supplementary material for: SpikeShip: A method for fast, unsupervised discovery of high-dimensional neural spiking patterns
Source: PLoS Comput Biol. 2023 Jul 31;19(7):e1011335. doi: 10.1371/journal.pcbi.1011335 (PMC10414626; doi:10.1371/journal.pcbi.1011335)
Supplement: S5 Fig — (A) Each pattern has a length of 300 samples, and is embedded in a larger window starting from -300 samples to +300 samples, with homogeneous noise surrounding the pattern on the left and right. The onset of the pattern is -150 samples plus some random offset Δtw. For each epoch realization, the value of Δtw was randomly chosen with uniform probability from an interval determined by the maximum window offset (max offset of 100 meant that Δtw ∈ [-100,100]). We select a window ranging from −Tw/2 to +Tw/2 samples of length Tw. (B) Clustering performance of 2D t-SNE embeddings was measured relative to ground-truth (ARI, compared with k-Means labels) and with an unsupervised performance measure, Silhouette Score. Clustering performance decreased as the maximum window offset increased, due to the inclusion of noise spikes around the spike pattern. SpikeShip has a small but consistent performance advantage relative to SPOTDis. Furthermore, SpikeShip strongly outperformed VP results in clustering performance, which as expected was severely distorted by global shifts in spiking patterns. ARI and Silhouette scores correspond to the mean value obtained across 10 repetitions for each combination of window length (Tw) and max window offset (Δtw). (PDF) [file pcbi.1011335.s005.pdf]

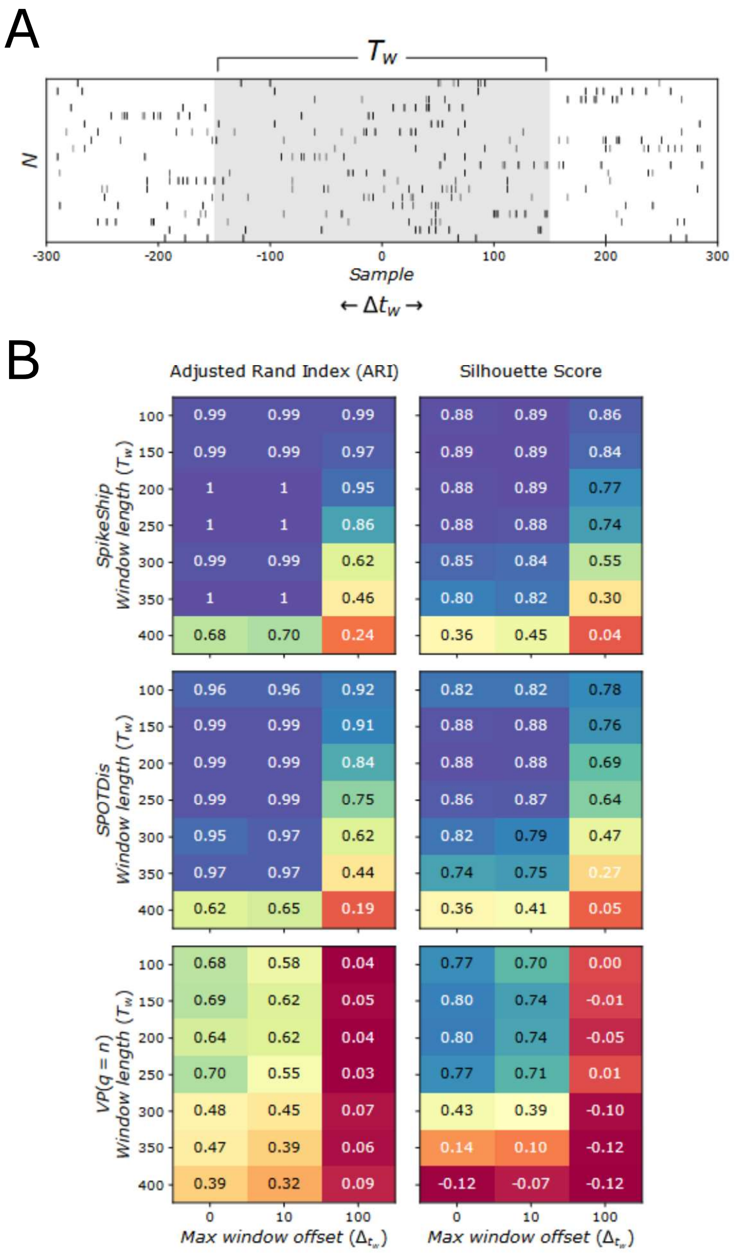


**Fig S5: Dependence of clustering performance on chosen window length and temporal jitter of spike pattern onset.** (A) Each pattern has a length of 300 samples, and is embedded in a larger window starting from -300 samples to +300 samples, with homogeneous noise surrounding the pattern on the left and right. The onset of the pattern is -150 samples plus some random offset $\Delta t_w$. For each epoch realization, the value of $\Delta t_w$ was randomly chosen with uniform probability from an interval determined by the maximum window offset (max offset of 100 meant that $\Delta t_w \in$ [-100,100]). We select a window ranging from $-T_w/2$ to $+T_w/2$ samples of length $T_w$. (B) Clustering performance of 2D t-SNE embeddings was measured relative to ground-truth (ARI, compared with k-Means labels) and with an unsupervised performance measure, Silhouette Score. Clustering performance decreased as the maximum window offset increased, due to the inclusion of noise spikes around the spike pattern. SpikeShip has a small but consistent performance advantage relative to SPOTDis. Furthermore, SpikeShip strongly outperformed VP results in clustering performance, which as expected was severely distorted by global shifts in spiking patterns. ARI and Silhouette scores correspond to the mean value obtained across 10 repetitions for each combination of window length ($T_w$) and max window offset ($\Delta t_w$).
