# Supplementary material for: SpikeShip: A method for fast, unsupervised discovery of high-dimensional neural spiking patterns
Source: PLoS Comput Biol. 2023 Jul 31;19(7):e1011335. doi: 10.1371/journal.pcbi.1011335 (PMC10414626; doi:10.1371/journal.pcbi.1011335)
Supplement: S6 Fig — We simulated 6 patterns with Poisson noise surrounding the pattern on the left and right. The onset of the pattern (i.e., Δtw) was randomly assigned between 0 and 0.8 with a window length of Tw = 0.2s for the patterns. The analysis window used here is 1 s, i.e. the entire period. SpikeShip correctly detects the 6 different patterns, but also SpikeShip can decompose the spike patterns to make it invariant to changes in global shifts. VP distance drastically depends on the global shift applied to the spikes. SpikeShip can retrieve the global shift from the spike sequences and reconstruct the random global shifts applied to the spike trains. (PDF) [file pcbi.1011335.s006.pdf]

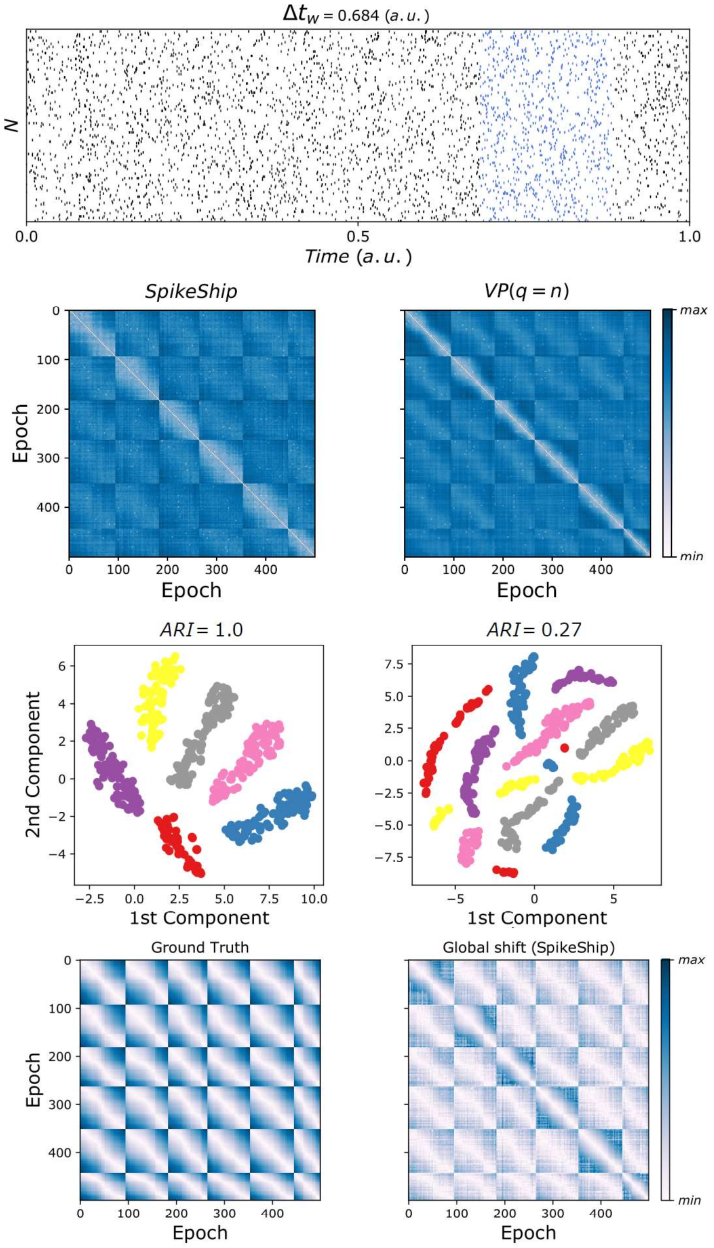

Fig S6: Comparison of VP and SpikeShip for simulations with multiple patterns and global shifts. We simulated 6 patterns with Poisson noise surrounding the pattern on the left and right. The onset of the pattern ($i.e., \Delta t_w$) was randomly assigned between 0 and 0.8 with a window length of $T_w = 0.2s$ for the patterns. The analysis window used here is 1 s, i.e. the entire period. SpikeShip correctly detects the 6 different patterns, but also SpikeShip can decompose the spike patterns to make it invariant to changes in global shifts. VP distance drastically depends on the global shift applied to the spikes. SpikeShip can retrieve the global shift from the spike sequences and reconstruct the random global shifts applied to the spike trains.
