# Supplementary material for: SpikeShip: A method for fast, unsupervised discovery of high-dimensional neural spiking patterns
Source: PLoS Comput Biol. 2023 Jul 31;19(7):e1011335. doi: 10.1371/journal.pcbi.1011335 (PMC10414626; doi:10.1371/journal.pcbi.1011335)
Supplement: S7 Fig — A) Global scaling. Same simulations as in S8 Fig. Victor-Purpura distance (VP) was used with different values of q. When q = 0, VP = |ni − nj|, with ni and nj the spike count of spike sequences i and j, respectively. Epochs are clustered based on rates. B) Local scaling. Same simulations as in S9 Fig. VP distance was used with different values of q. When q → ∞, VP = ni + nj. Besides high values of q aim to extract temporal information from spike trains, these 2D embeddings demonstrate that the contribution between rate and timing using VP is difficult to interpret and very sensitive to noise. (PDF) [file pcbi.1011335.s007.pdf]

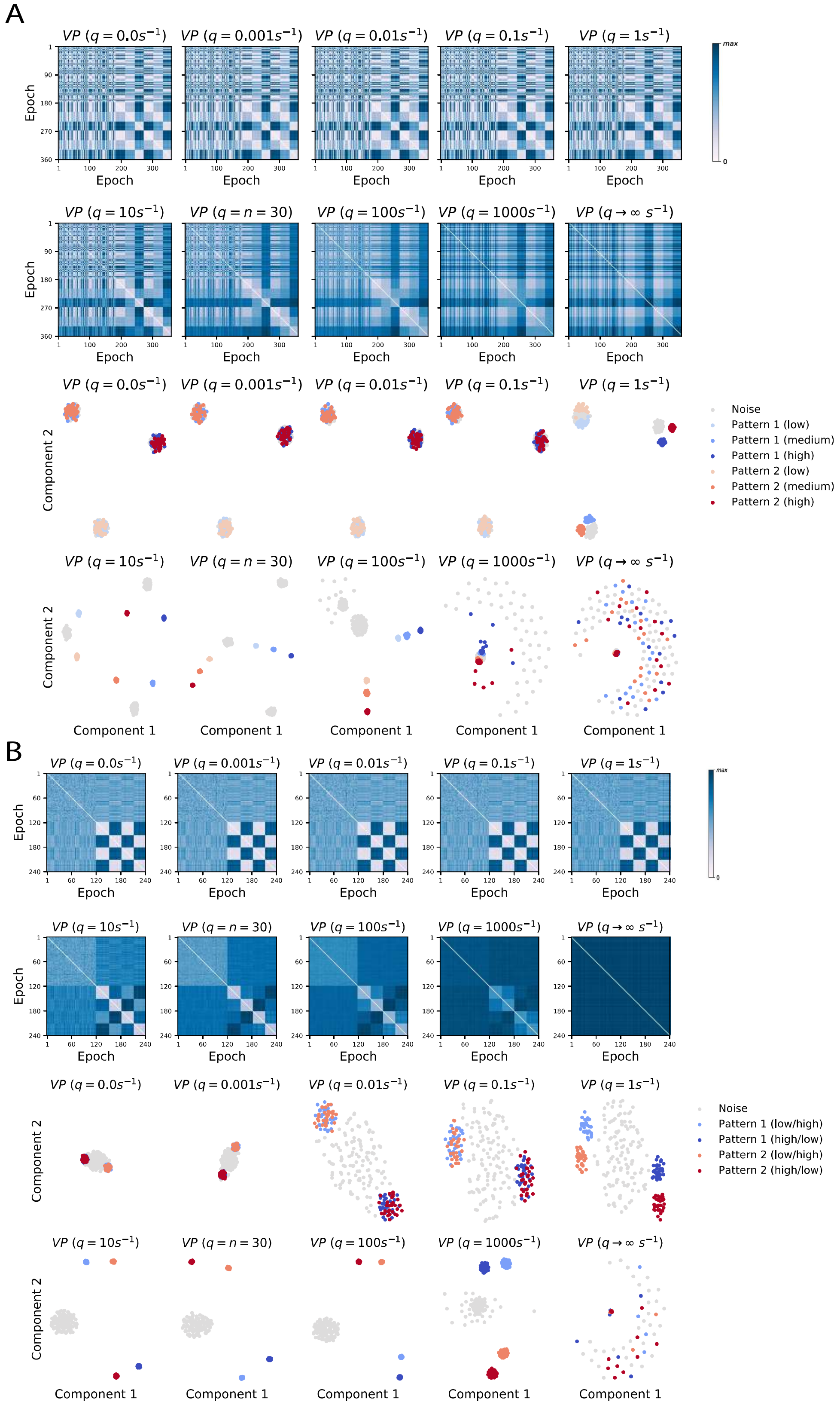

Fig S7: Performance of VP distance is affected by changes in both local and global scaling rates. A) Global scaling. Same simulations as in S8 Fig. Victor-Purpura distance (VP) was used with different values of q. When $q = 0$, $VP = |n_i - n_j|$, with n_i and n_j the spike count of spike sequences i and j, respectively. Epochs are clustered based on rates. B) Local scaling. Same simulations as in S9 Fig. VP distance was used with different values of q. When $q \to \infty$, $VP = n_i + n_j$. Besides high values of q aim to extract temporal information from spike trains, these 2D embeddings demonstrate that the contribution between rate and timing using VP is difficult to interpret and very sensitive to noise.
