# Supplementary material for: SpikeShip: A method for fast, unsupervised discovery of high-dimensional neural spiking patterns
Source: PLoS Comput Biol. 2023 Jul 31;19(7):e1011335. doi: 10.1371/journal.pcbi.1011335 (PMC10414626; doi:10.1371/journal.pcbi.1011335)
Supplement: S8 Fig — Two different temporal patterns with different firing rates. Each temporal pattern can occur in a low (λin = 0.2 and λout = 0.02 spks/sample), medium (λin = 0.4 and λout = 0.04 spks/sample) or high rate (λin = 0.7 and λout 0.07 spks/sample) state, with a constant ratio of λin/λout. In addition, the noise pattern can also occur in one of three rate states. The pulse duration was 30 samples. Shown at the bottom the sorted dissimilarity matrix with SpikeShip values, the t-SNE embedding with the ground-truth cluster labels and the t-SNE embedding with the HDBSCAN cluster labels. (PDF) [file pcbi.1011335.s008.pdf]

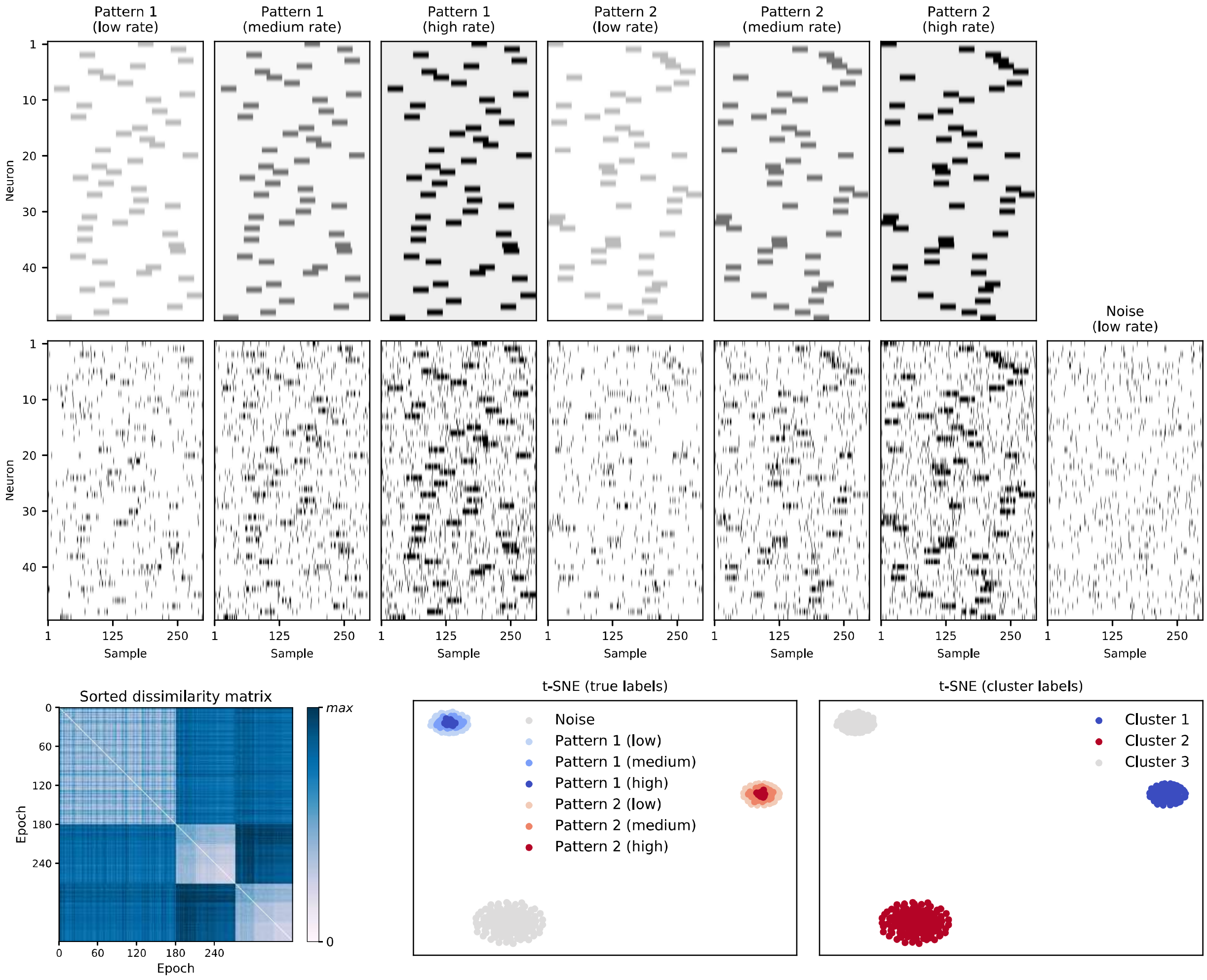


**Fig S8: Performance of SpikeShip is not affected by a global scaling rate.** We defined $\lambda_{in}$ and $\lambda_{out}$ as the firing rates of simulated patterns and the baseline firing rate, respectively (measured in spikes/sample). Shown are two different temporal patterns. Each temporal pattern can occur in a low ($\lambda_{in} = 0.2$ and $\lambda_{out} = 0.02$ spks/sample), medium ($\lambda_{in} = 0.4$ and $\lambda_{out} = 0.04$ spks/sample) or high rate ($\lambda_{in} = 0.7$ and $\lambda_{out} = 0.07$ spks/sample) state, with a constant ratio of $\lambda_{in}/\lambda_{out}$. In addition, the noise pattern can also occur in one of three rate states. The pulse duration was 30 samples. Shown at the bottom is the sorted dissimilarity matrix with SpikeShip values, the t-SNE embedding with the ground-truth cluster labels and the t-SNE embedding with the HDBSCAN cluster labels.
