# Supplementary material for: SpikeShip: A method for fast, unsupervised discovery of high-dimensional neural spiking patterns
Source: PLoS Comput Biol. 2023 Jul 31;19(7):e1011335. doi: 10.1371/journal.pcbi.1011335 (PMC10414626; doi:10.1371/journal.pcbi.1011335)
Supplement: S9 Fig — Two different temporal patterns with different firing rates. Each temporal pattern could occur in one of two rates states: In the first rate state, the first 25 neurons are firing at a low rate (λin = 0.3 and λout = 0.03 spks/sample), and the other 25 are firing at a high rate (λin = 0.7 and λout = 0.07 spks/sample). In the second rate state, the rate scaling is reversed. The pulse duration was 30 samples. Shown at the bottom the sorted dissimilarity matrix with SpikeShip values, the t-SNE embedding with the ground-truth cluster labels and the t-SNE embedding with the HDBSCAN cluster labels. (PDF) [file pcbi.1011335.s009.pdf]

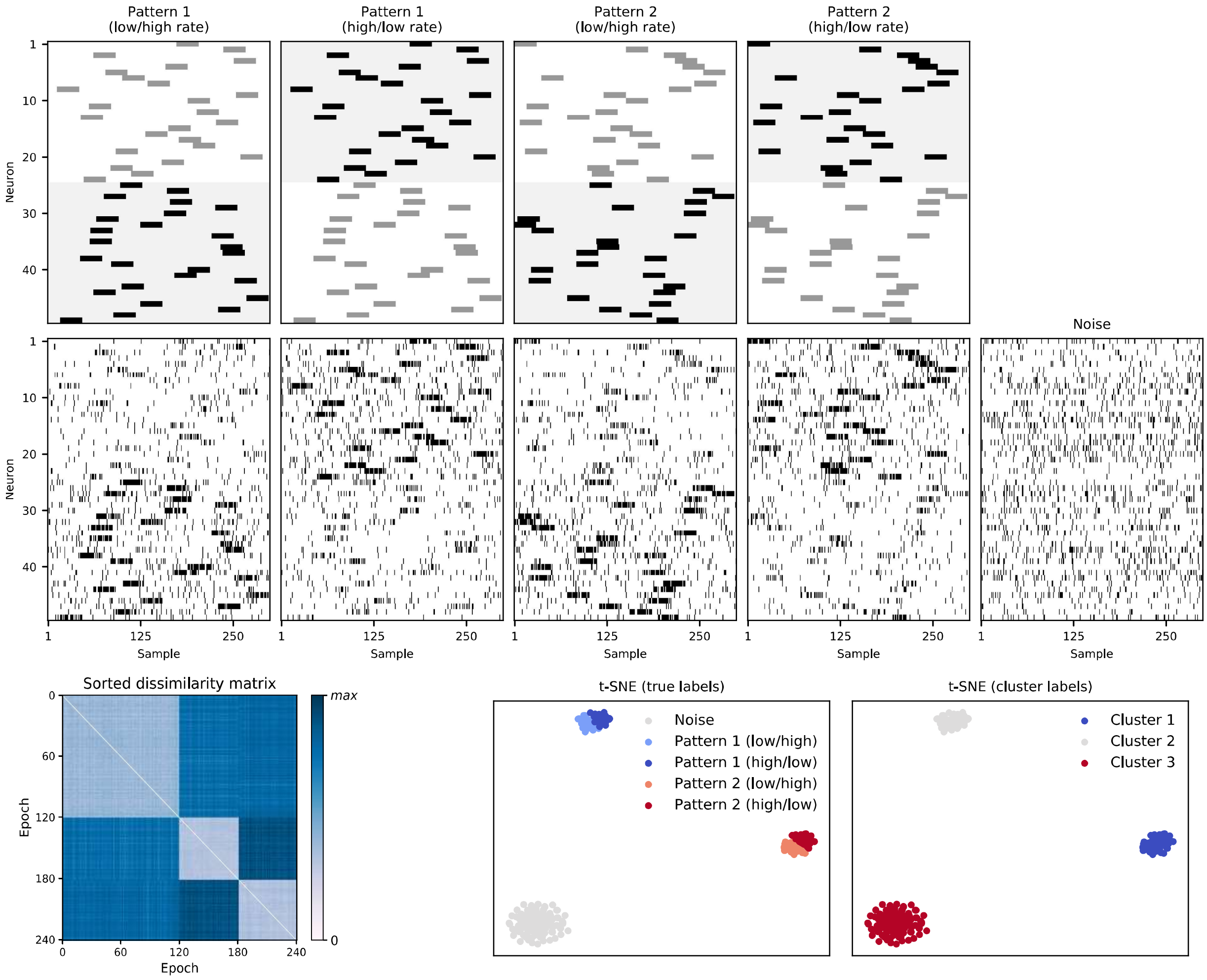

Fig S9: Performance of SpikeShip is not affected by a local scaling rate. We defined λ_{in} and λ_{out} as the firing rates of simulated patterns and the baseline firing rate, respectively (measured in spikes/sample). Shown are two temporal patterns. Each temporal pattern could occur in one of two rates states: In the first rate state, the first 25 neurons are firing at a low rate ($\lambda_{in} = 0.3$ and $\lambda_{out} = 0.03$ spks/sample), and the other 25 are firing at a high rate ($\lambda_{in} = 0.7$ and $\lambda_{out} = 0.07$ spks/sample). In the second rate state, the rate scaling is reversed. The pulse duration was 30 samples. Shown at the bottom the sorted dissimilarity matrix with SpikeShip values, the t-SNE embedding with the ground-truth cluster labels and the t-SNE embedding with the HDBSCAN cluster labels.
