# Supplementary material for: SpikeShip: A method for fast, unsupervised discovery of high-dimensional neural spiking patterns
Source: PLoS Comput Biol. 2023 Jul 31;19(7):e1011335. doi: 10.1371/journal.pcbi.1011335 (PMC10414626; doi:10.1371/journal.pcbi.1011335)
Supplement: S10 Fig — A) Global scaling. SPIKE and RI-SPIKE computations for globally scaled sequences. Top: dissimilarity matrices sorted by pattern id and scaling factor. Bottom: 2D t-SNE embeddings of epochs. B) Local scaling. SPIKE and RI-SPIKE computations for locally scaled sequences. Both A and B were computed using the same simulations as in S8 Fig. Top: dissimilarity matrices sorted by pattern id and scaling factor. Bottom: 2D t-SNE embeddings of epochs. The first 180 epochs correspond to noise. (PDF) [file pcbi.1011335.s010.pdf]

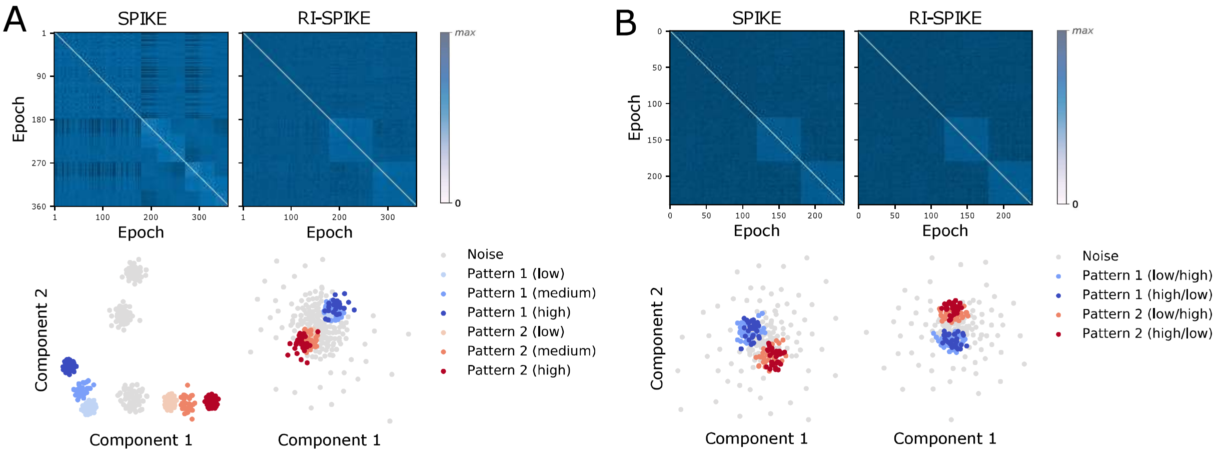


**Fig S10: Performance of SPIKE and RI-SPIKE are affected by both global and local scaling.** A) Global scaling. SPIKE and RI-SPIKE computations for globally scaled sequences. Top: dissimilarity matrices sorted by pattern id and scaling factor. Bottom: 2D t-SNE embeddings of epochs. B) Local scaling. SPIKE and RI-SPIKE computations for locally scaled sequences. Both A and B were computed using the same simulations as in S8 Fig. Top: dissimilarity matrices sorted by pattern id and scaling factor. Bottom: 2D t-SNE embeddings of epochs. The first 180 epochs correspond to noise.
