# Supplementary material for: SpikeShip: A method for fast, unsupervised discovery of high-dimensional neural spiking patterns
Source: PLoS Comput Biol. 2023 Jul 31;19(7):e1011335. doi: 10.1371/journal.pcbi.1011335 (PMC10414626; doi:10.1371/journal.pcbi.1011335)
Supplement: S11 Fig — A) Pairwise comparison of epochs and clustering performance of metrics (one session). Top: Dissimilarity matrices sorted by Natural scene ID. Bottom: 2D t-SNE embeddings. Clustering performance was computed via Adjusted Rank Index (ARI). We used Gaussian Mixture (GM) to assign labels to each cluster. B) Distribution of clustering performances (ARIGM) across sessions. For VP distances, the average number of spike times (n) was computed for every session. The mean and standard deviation of n and N across all the sessions were (μn = 2.1, σn = 0.26) and (μN = 570.4, σN = 190.6), respectively. Median values of ARIGM for Firing rates, RI-SPIKE, and SpikeShip are 0.1848, 0.075, and 0.1865, respectively. We performed the Welch’s t-test across epochs. The obtained p-values from comparison of SpikeShip and all the other metrics were: (Firing Rates, SpikeShip) = 6.71 × 10^−2^, (RI-SPIKE, SpikeShip) = 5.49 × 10^−9^. (PDF) [file pcbi.1011335.s011.pdf]

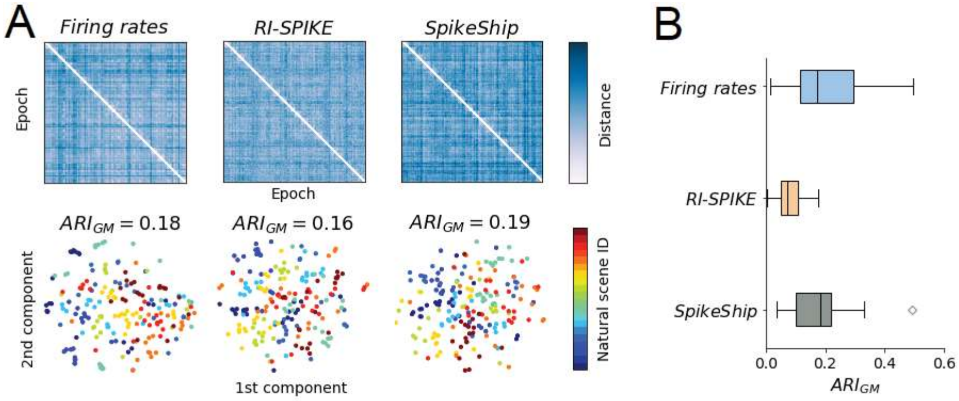

Fig S11: Comparison of spike train metrics across single sessions during Natural Scene presentations. A) Pairwise comparison of epochs and clustering performance of metrics (one session). Top: Dissimilarity matrices sorted by Natural scene ID. Bottom: 2D t-SNE embeddings. Clustering performance was computed via Adjusted Rank Index (ARI). We used Gaussian Mixture (GM) to assign labels to each cluster. B) Distribution of clustering performances (ARI_{GM}) across sessions. For VP distances, the average number of spike times (n) was computed for every session. The mean and standard deviation of n and N across all the sessions were ($\mu_n = 2.1, \sigma_n = 0.26$) and ($\mu_N = 570.4, \sigma_N = 190.6$), respectively. Median values of ARI_{GM} for Firing rates, RI-SPIKE, and SpikeShip are 0.1848, 0.075, and 0.1865, respectively. We performed the Welch's t-test across epochs. The obtained p-values from comparison of SpikeShip and all the other metrics were: (Firing Rates, SpikeShip) $= 6.71 \times 10^{-2}$, (RI-SPIKE, SpikeShip) $= 5.49 \times 10^{-9}$.
