# Supplementary material for: SpikeShip: A method for fast, unsupervised discovery of high-dimensional neural spiking patterns
Source: PLoS Comput Biol. 2023 Jul 31;19(7):e1011335. doi: 10.1371/journal.pcbi.1011335 (PMC10414626; doi:10.1371/journal.pcbi.1011335)
Supplement: S12 Fig — A) 2D t-SNE embeddings from SpikeShip and Firing rates’ dissimilarity matrices. The allocation of natural scenes’ clusters are different between the two embeddings. Natural scenes are represented by their ID. B) Scaled Euclidean pairwise distance between centroids of each cluster for both SpikeShip (Left) and firing rates (Middle), and their difference (Right). (PDF) [file pcbi.1011335.s012.pdf]

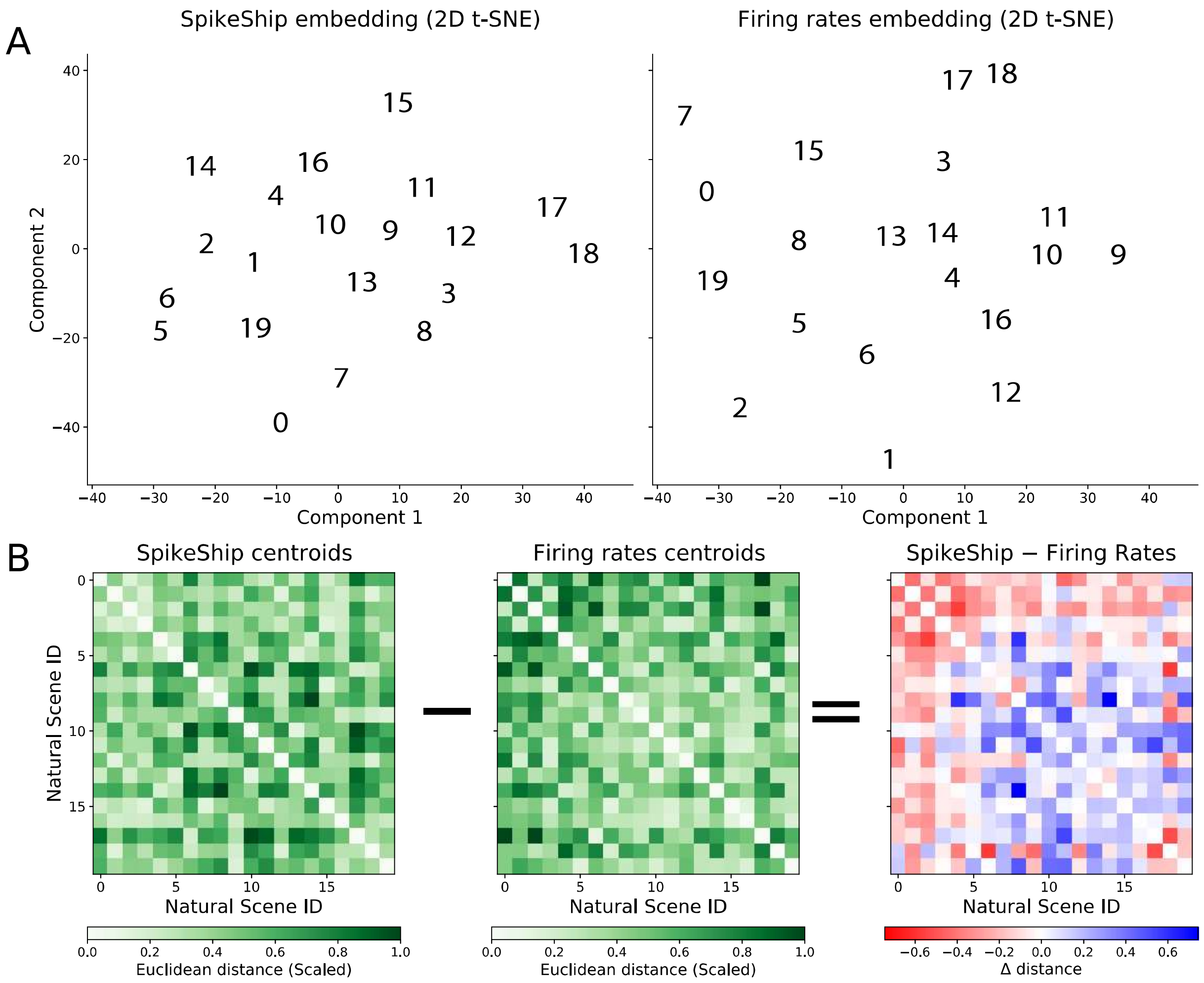

Fig S12: Comparison between clusters from SpikeShip and Firing rates embeddings of Natural scenes. A) 2D t-SNE embeddings from SpikeShip and Firing rates' dissimilarity matrices. The allocation of natural scenes' clusters are different between the two embeddings. Natural scenes are represented by their ID. B) Scaled Euclidean pairwise distance between centroids of each cluster for both SpikeShip (Left) and firing rates (Middle), and their difference (Right).
