# Supplementary material for: SpikeShip: A method for fast, unsupervised discovery of high-dimensional neural spiking patterns
Source: PLoS Comput Biol. 2023 Jul 31;19(7):e1011335. doi: 10.1371/journal.pcbi.1011335 (PMC10414626; doi:10.1371/journal.pcbi.1011335)
Supplement: S13 Fig — Top: Dissimilarity matrices sorted by Natural Scene ID. Middle: 2D t-SNE embeddings from dissimilarity matrices colored by Natural Scene ID. Bottom: The clustering performance through ARI score and Spearman correlation between dissimilarity matrices computed via SPIKE, RI-SPIKE, Firing rates, and SpikeShip. The clustering performance of SPIKE and RI-SPIKE is lower than the clustering performance by using the traditional firing rates and SpikeShip. SPIKE and RI-SPIKE are highly correlated while Firing Rates and SpikeShip are highly uncorrelated. (PDF) [file pcbi.1011335.s013.pdf]

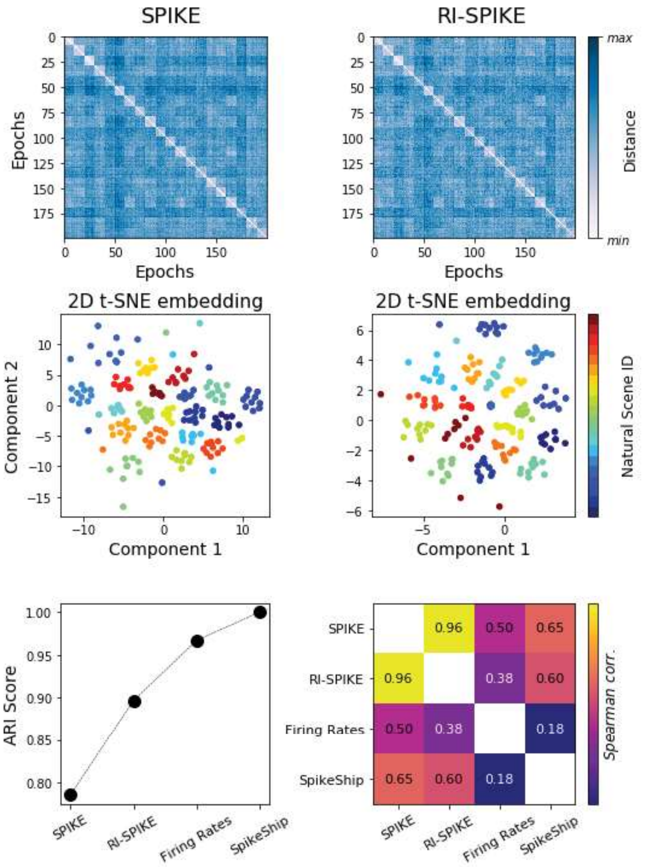

Fig S13: Analysis of large scale neural recordings during visual stimuli presentations with SPIKE and RI-SPIKE. Top: Dissimilarity matrices sorted by Natural Scene ID. Middle: 2D t-SNE embeddings from dissimilarity matrices colored by Natural Scene ID. Bottom: The clustering performance through ARI score and Spearman correlation between dissimilarity matrices computed via SPIKE, RI-SPIKE, Firing rates, and SpikeShip. The clustering performance of SPIKE and RI-SPIKE is lower than the clustering performance by using the traditional firing rates and SpikeShip. SPIKE and RI-SPIKE are highly correlated while Firing Rates and SpikeShip are highly uncorrelated.
