# Supplementary material for: SpikeShip: A method for fast, unsupervised discovery of high-dimensional neural spiking patterns
Source: PLoS Comput Biol. 2023 Jul 31;19(7):e1011335. doi: 10.1371/journal.pcbi.1011335 (PMC10414626; doi:10.1371/journal.pcbi.1011335)
Supplement: S14 Fig — Multi-spike sequence analyses for three mice (rows). Left: dissimilarity matrices. Middle: 2D t-SNE embedding. Right: 2D Spectral Embedding (Laplacian Eigenmaps). (PDF) [file pcbi.1011335.s014.pdf]

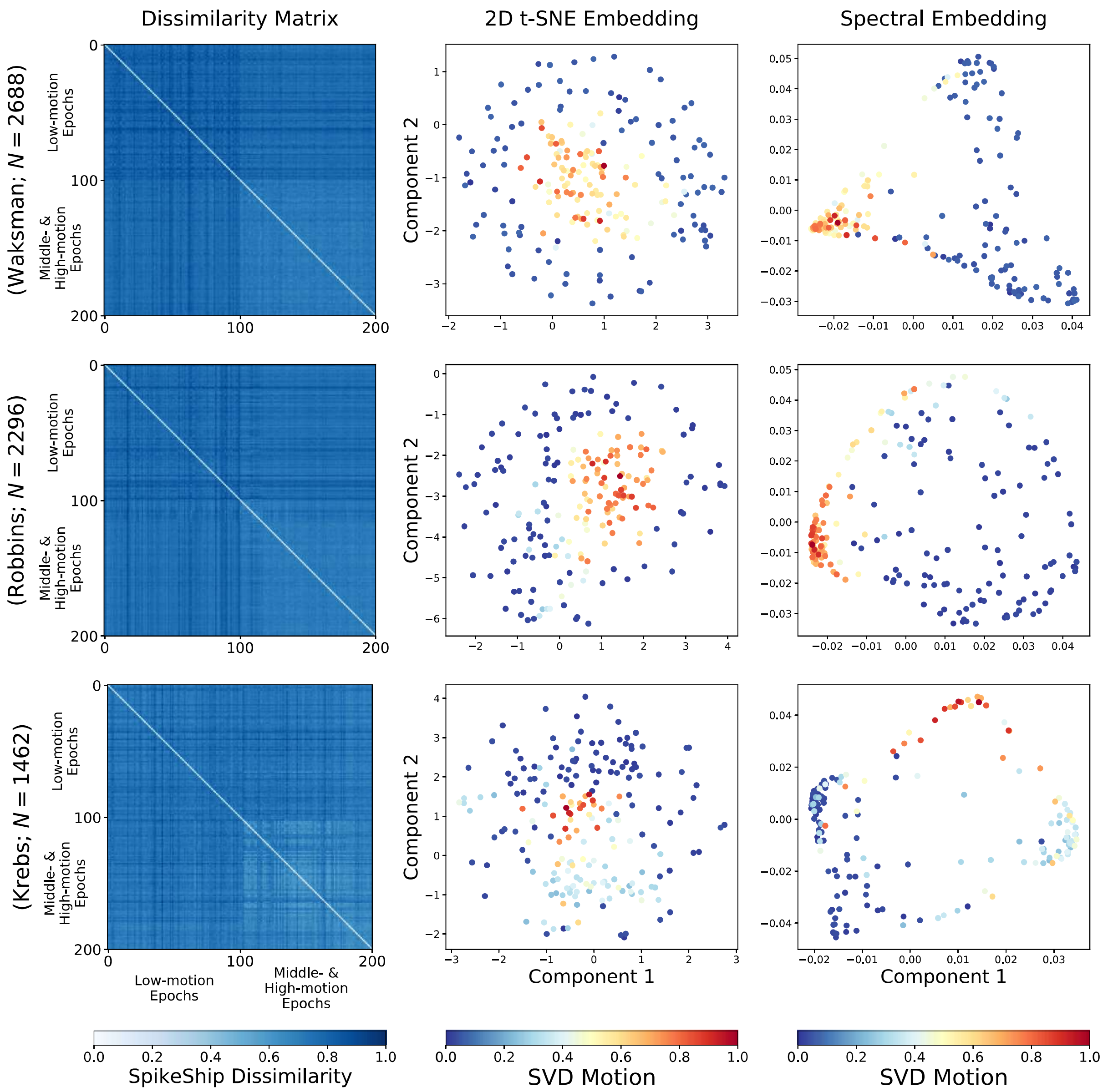

Fig S14: Spontaneous activity analyses for 3 mice. Multi-spike sequence analyses for three mice (rows). Left: dissimilarity matrices. Middle: 2D t-SNE embedding. Right: 2D Spectral Embedding (Laplacian Eigenmaps).
