# Supplementary material for: SpikeShip: A method for fast, unsupervised discovery of high-dimensional neural spiking patterns
Source: PLoS Comput Biol. 2023 Jul 31;19(7):e1011335. doi: 10.1371/journal.pcbi.1011335 (PMC10414626; doi:10.1371/journal.pcbi.1011335)
Supplement: S15 Fig — Normalized distribution of L2-distances between each epoch to the centroid for t-SNE and Spectral embedding (SE). We performed the Welch’s t-test between low-motion and middle- & high-motion epochs (variances between two groups were smaller than 5 × 10^−4^). Thus, (*) if p-value < 0.005, (**) if p-value < 0.05, and (***) if p-value < 0.5 (i.e., evidence against null hypothesis of equal population means). (PDF) [file pcbi.1011335.s015.pdf]

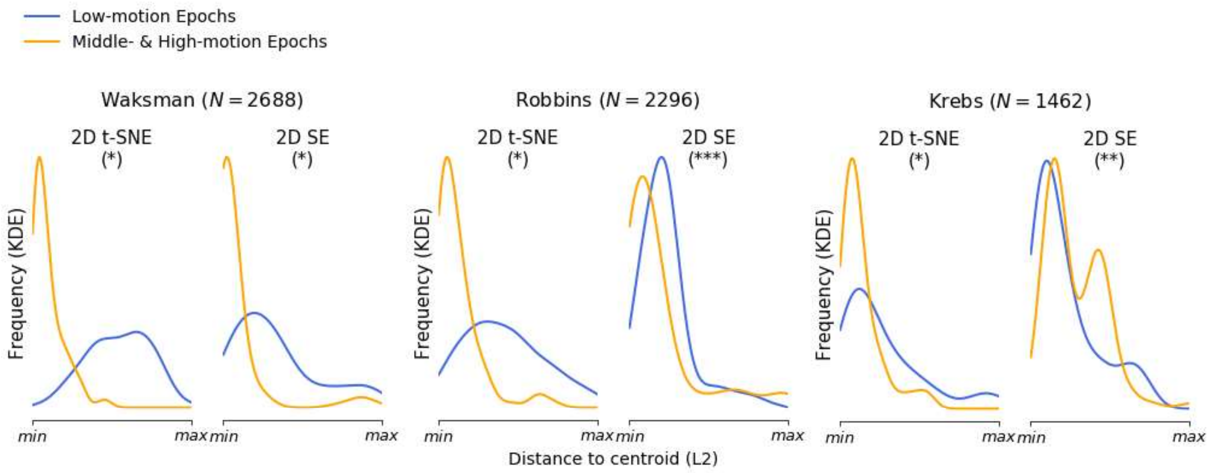

Fig S15: Variability of epochs allows distinction between low-motion and middle- & high-motion epochs. Normalized distribution of L2-distances between each epoch to the centroid for t-SNE and Spectral embedding (SE). We performed the Welch's t-test between low-motion and middle- & high-motion epochs (variances between two groups were smaller than 5×10^{-4}). Thus, (*) if $p\text{-}value < 0.005$, (**) if $p\text{-}value < 0.05$, and (***) if $p\text{-}value < 0.5$ (i.e., evidence against null hypothesis of equal population means).
