# Supplementary material for: SpikeShip: A method for fast, unsupervised discovery of high-dimensional neural spiking patterns
Source: PLoS Comput Biol. 2023 Jul 31;19(7):e1011335. doi: 10.1371/journal.pcbi.1011335 (PMC10414626; doi:10.1371/journal.pcbi.1011335)
Supplement: S16 Fig — Simulations of spike trains with same number of spikes (i.e., n = 20). Top: Comparison of distances between VP and EMD. Spike trains were defined as tm = (0, …, 0) and tk = (T, …, T), where T is the window length. Left: VP(q = 1/n) reaches a maximum value until T = 2n = 40. After that threshold, VP(q = 1/n) assign insertion costs rather than shifts. Right: (T/n) VP(q = 1/T) equals EMD. Bottom: Comparison of the average distances between VP and EMD across 100 simulations. Spike times for tm and tk (with same spike count) were randomly generated from a uniform distribution U(0, T). (PDF) [file pcbi.1011335.s016.pdf]

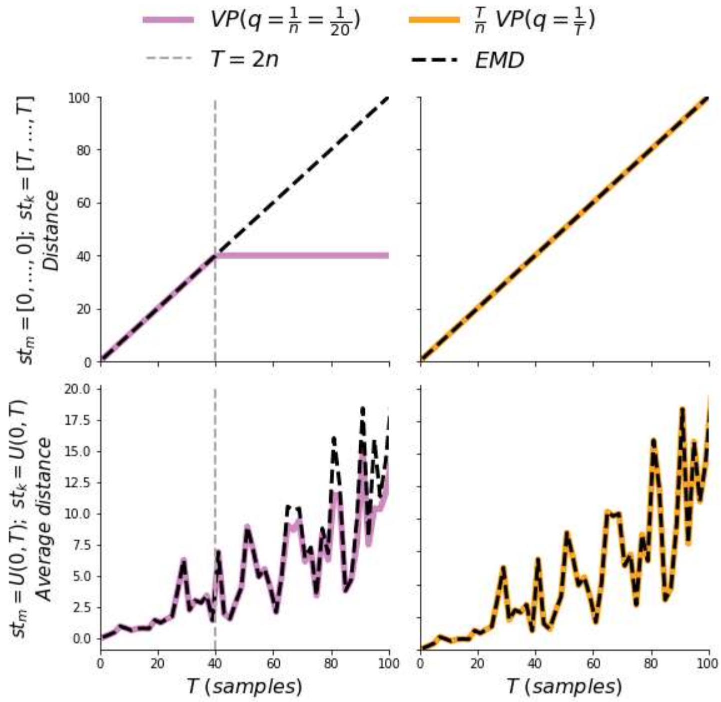

Fig S16: Relation between VP and EMD distances for spike trains with same spike counts. Simulations of spike trains with same number of spikes (i.e., $n = 20$). Top: Comparison of distances between VP and EMD. Spike trains were defined as $t_m = (0, \dots, 0)$ and $t_k = (T, \dots, T)$, where T is the window length. Left: VP($q = 1/n$) reaches a maximum value until $T = 2n = 40$. After that threshold, VP($q = 1/n$) assign insertion costs rather than shifts. Right: (T/n) VP($q = 1/T$) equals EMD. Bottom: Comparison of the average distances between VP and EMD across 100 simulations. Spike times for t_m and t_k (with same spike count) were randomly generated from a uniform distribution $U(0, T)$.
